# Recent Developments in Vinylsulfonium and Vinylsulfoxonium Salt Chemistry

**DOI:** 10.3390/molecules23040738

**Published:** 2018-03-23

**Authors:** Mukulesh Mondal, Shi Chen, Nessan J. Kerrigan

**Affiliations:** 1 Department of Chemistry, Oakland University, Rochester, MI 40309, USA; mondal@oakland.edu (M.M.); chenshix@yahoo.com (S.C.); 2 School of Chemical Sciences, Dublin City University, Glasnevin, Dublin 9, Ireland

**Keywords:** vinylsulfonium, vinylsulfoxonium, sulfonium salts, sulfoxonium salts, oxosulfonium, ylides, conjugate addition, epoxide, cyclopropane, *γ*-lactone

## Abstract

This review describes advances in the literature since 2000 in the area of reactions of vinylsulfonium and vinylsulfoxonium salts, with a particular emphasis on stereoselective examples. Although the chemistry of vinylsulfonium salts was first explored back in the 1950s, and that of vinylsulfoxonium salts in the early 1970s, there has been renewed interest in these compounds since the turn of the century. This has been largely due to an increased appreciation for the many synthetic possibilities associated with these valuable electrophiles. The development of improved routes to vinylsulfonium salts allowing for their in situ generation has played a part in accelerating their study. In general, reactions of the two sulfur salt classes follow a similar mechanistic pathway: initial conjugate addition of a nucleophile to the *β*-position, followed by protonation of an ylide intermediate, and cyclization of tethered anion to afford monocyclic or bicyclic product (e.g., cyclopropane, aziridine, oxazole, oxazolidinone, *γ*-lactam or *γ*-lactone). Alternatively, reactions involve formation of an ylide intermediate followed by intramolecular Johnson-Corey-Chaykovsky reaction (epoxidation or cyclopropanation), and subsequent cyclization to afford the desired bicyclic product (e.g., fused bicyclic epoxide or cyclopropane).

## 1. Introduction

Ever since the independent contributions of Johnson, Corey and Chaykovsky in the 1960s, organosulfur chemistry has assumed a central position in synthetic organic chemistry [1,2]. This is mainly because sulfur ylide and sulfur salt chemistry has provided simple, efficient, and frequently highly stereoselective (diastereoselective and enantioselective) routes to important synthetic building blocks, including epoxides, cyclopropanes, aziridines, and more recently *γ*-lactones and alkenes. These reaction products often function as key intermediates in natural product and in pharmaceutical synthesis, and so have broad appeal to both academia and to industry. This review will center on recent contributions to the literature on chemistry of vinyl sulfonium and vinyl sulfoxonium salts since 2000, as prior to this review a number of excellent treatises describing progress in the area of sulfur ylide and sulfur salt chemistry have been presented [3,4,5,6,7].

## 2. Sulfur is Special

The sulfur atom’s ability to stabilize an adjacent (*α*) negatively charged carbon has long attracted attention from synthetic organic and theoretical chemists [4,5,6,7]. This stabilization was at one point attributed to the ability of empty low-lying d-orbitals to delocalize electron density from the filled orbital of the carbanion [8]. However, more recently ab initio computational studies have shed more light on the mode of stabilization. It is now generally accepted that delocalization into d-orbitals does not play a role in stabilization of sulfur ylides [4,5,9,10,11]. Instead, it has been proposed that the stabilization is largely due to electrostatic attraction, and negative hyperconjugation (overlap between the carbanion lone pair orbital *n* with the σ* orbital of sulfur-carbon bond) [9,10,11,12,13,14]. Therefore, it is not surprising that vinylsulfonium and vinylsulfoxonium salts function as effective electrophilic Michael acceptors akin to *α*,*β*-unsaturated ketones, given the ability of the sulfur atom to stabilize ylide intermediates (sulfonium and sulfoxonium ylides) and associated transition states. In contrast, the analogous vinyl ammonium salts do not act as Michael acceptors [6,7].

## 3. Early Contributions

In 1966 Gosselck and co-workers demonstrated that cyclopropanes could be assembled through the reaction of carbanions with vinyldimethylsulfonium salts [15,16,17,18]. Shortly after, in the early 1970s, Johnson’s group advanced the idea of vinylsulfoxonium salts as electrophilic species which could potentially provide a new arsenal of reactions that would complement those that had been developed using various nucleophilic sulfur ylides [19,20]. In 1980 Johnson’s group demonstrated that vinylsulfoxonium salts could undergo conjugate addition reactions with a range of protic nucleophiles (some ambident), including amines, ketoesters, and nitromethane to afford 3-membered and 5-membered cyclic products [21]. However, vinylsulfoxonium salt research remained largely dormant until Gais’s group carried out further work in the 1990s and 2000s [22,23,24]. On the other hand, vinylsulfoniums have attracted a lot of interest recently, in no small part, due to the contributions of the Aggarwal group in the period 2000 to present [25]. This review will focus on recent contributions to the literature on the chemistry of vinylsulfonium and vinylsulfoxonium salts since 2000.

## 4. Synthesis of Vinylsulfonium and Vinylsulfoxonium Salts

### 4.1. Synthesis of Diphenylvinylsulfonium Triflate *(**1**)*

**Scheme 1:**
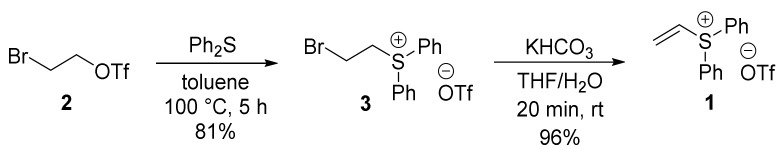
Synthesis of diphenylvinylsulfonium triflate.

Aggarwal and colleagues recently reported an efficient and practical method for the synthesis of diphenylvinylsulfonium triflate (Scheme 1) [25,26]. The reaction of 2-bromoethyl triflate **2** with diphenyl sulfide in refluxing toluene under an inert atmosphere furnished crystalline solid bromoethyldiphenylsulfonium triflate **3** in high yield. Bromoethyldiphenylsulfonium triflate **3** on treatment with potassium bicarbonate in a mixture of tetrahydrofuran and water at room temperature gave the desired diphenylvinylsulfonium triflate **1** as a light-yellow oil in excellent yield. Bromoethyldiphenylsulfonium triflate **3** is now commercially available and used as a precursor for the in-situ generation of diphenylvinylsulfonium triflate under various reaction conditions.

### 4.2. Synthesis of Vinylsulfoxonium Salts *(**16**)*

**Scheme 2:**
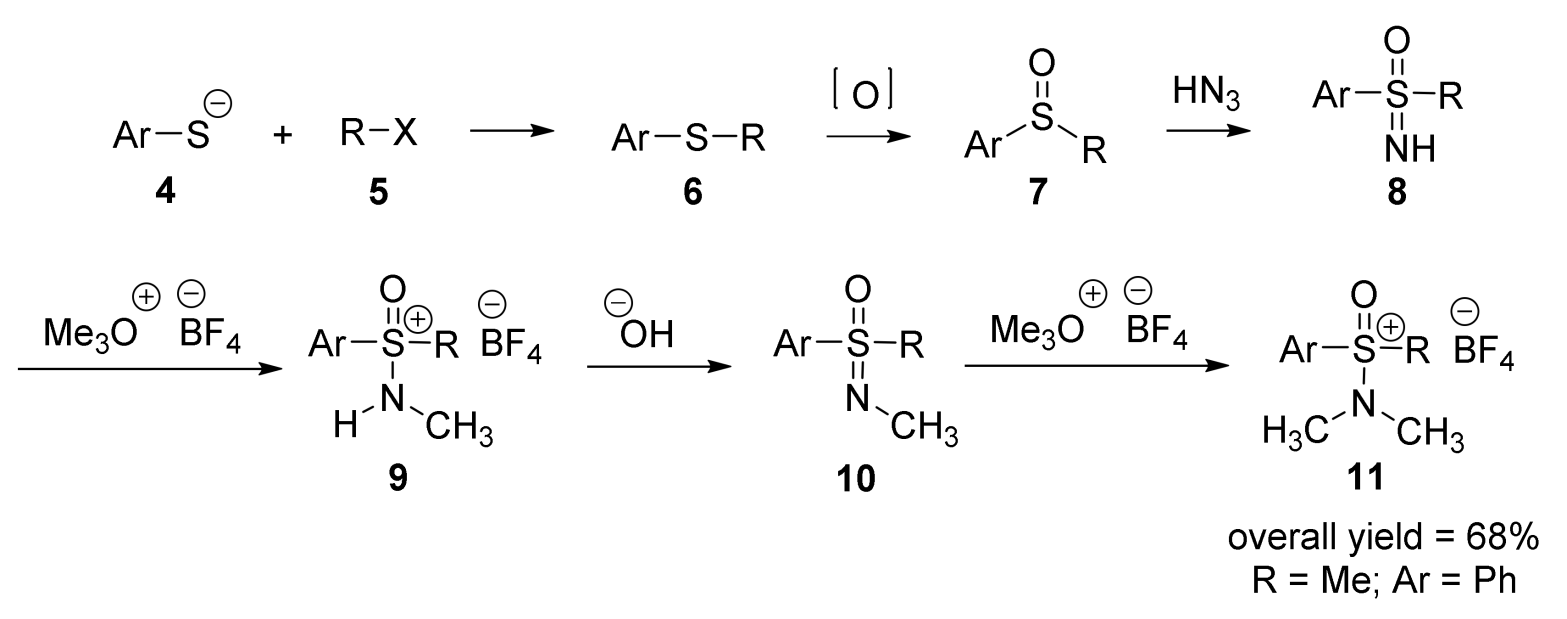
Preparation of dialkylaminosulfoxonium salts.

Johnson and co-workers reported the first synthesis of aminosulfoxonium salts **11** (Scheme 2) [27]. The preparation started with the alkylation of an arenethiolate, followed by a smooth oxidation of the resulting sulfide to a sulfoxide, then a conversion of the corresponding sulfoxide to a sulfoximine. It was determined that hydrazoic acid gave a high yield (92%) for the latter conversion in the case of methylphenylsulfoxide. Alkylation of free sulfoximine to dimethylaminosulfoxonium fluoroborate was completed by employing Meerwein’s salt (trimethyloxonium tetrafluoroborate) as methylating agent. Exhaustive alkylation produced the desired dimethylaminosulfoxonium salt **11**, while attempted monoalkylation usually resulted in a mixture of products (**9** and **11**).

**Scheme 3:**
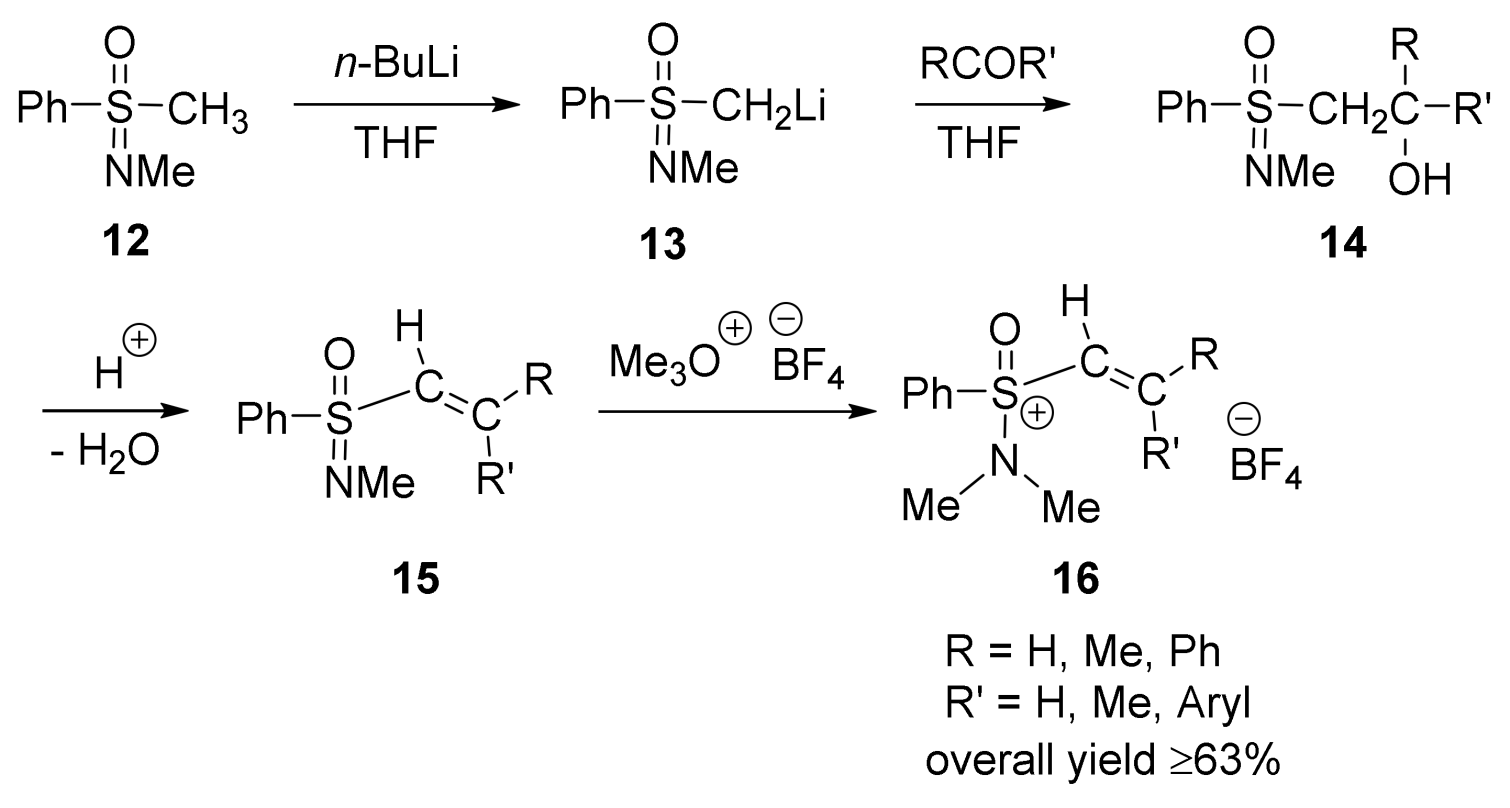
Synthesis of vinylsulfoxonium salts.

Following their procedure for the synthesis of dialkylaminosulfoxonium salts, Johnson and co-workers were able to isolate and characterize mono-*N*-alkyl sulfoximines **10** [27]. Deprotonation of the sulfoximine with a strong base, and then addition to an aldehyde or ketone to afford **14**, followed by dehydration under acidic conditions furnished *α*,*β*-unsaturated sulfoximine **15**. Treatment of **14** with concentrated sulfuric acid at 0 °C gave excellent yield (≥96%) for the dehydration step after an examination of different reaction conditions. Alternatively, mesylation of **14** followed by DBU-mediated elimination provided **15** with high olefin isomer stereoselectivity [28]. Finally, methylation of **15** with trimethyloxonium tetrafluoroborate in dichloromethane yielded the desired vinylsulfoxonium salt **16** in high purity (Scheme 3) [19,20,21,27].

## 5. Reactions of Vinylsulfonium Salts

**Scheme 4:**
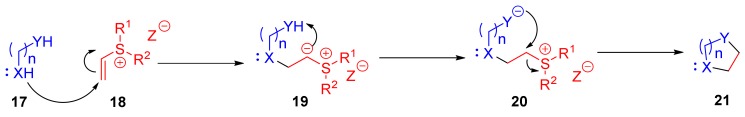
Pathway 1.

**Scheme 5:**
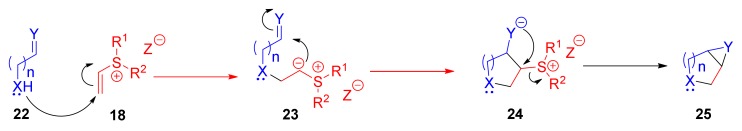
Pathway 2.

Vinylsulfonium salts are generally very reactive towards conjugate addition by various nucleophiles, thereby producing sulfur ylide intermediates. There are two possible pathways through which the newly generated sulfonium ylide can react [25,26]. In the first pathway, the sulfonium ylide **19**, generated by conjugate addition of a suitable nucleophile, undergoes intramolecular protonation from a neighboring acidic site to generate a new nucleophilic center. The second nucleophile facilitates ring closure with the displacement of the sulfonium leaving group (Scheme 4) [29]. The second pathway is more similar to Johnson–Corey–Chaykovsky-type reactions, where the ylide intermediate **23** undergoes intramolecular 1,2- or 1,4-addition to a pendant electrophile (aldehyde, ketone, imine, or Michael acceptor) to generate a new zwitterionic intermediate **24**. The zwitterionic intermediate **24** undergoes ring closure with the displacement of sulfide to produce fused heterocycles **25** (Scheme 5) [30,31,32].

### 5.1. Three-Membered Heterocycle Synthesis

**Scheme 6:**
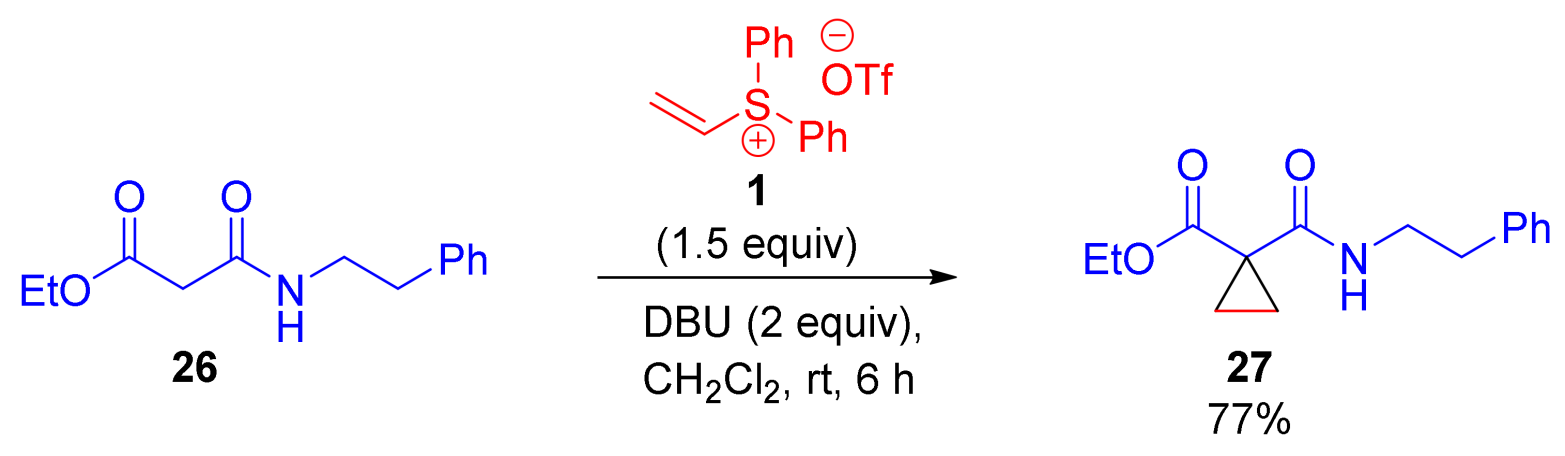
Cyclopropanation of malonyl amide.

Compounds with an activated methylene group react with vinyl sulfonium salts in the presence of base to generate ylide intermediates. The ylides on subsequent proton transfer followed by ring closure, with the displacement of sulfide, produce cyclopropane derivatives. Xie and colleagues reported the synthesis of gem-disubstituted cyclopropanes **27** from the reaction of *N*-alkyl malonyl amides **26** and diphenylvinylsulfonium triflate **1** in the presence of the organic base DBU under mild conditions and with good yields (Scheme 6) [33]. Similarly, reactions of primary amines and sulfonamides with vinylsulfonium salt **1** in the presence of mild base furnished aziridines [34,35].

**Scheme 7:**
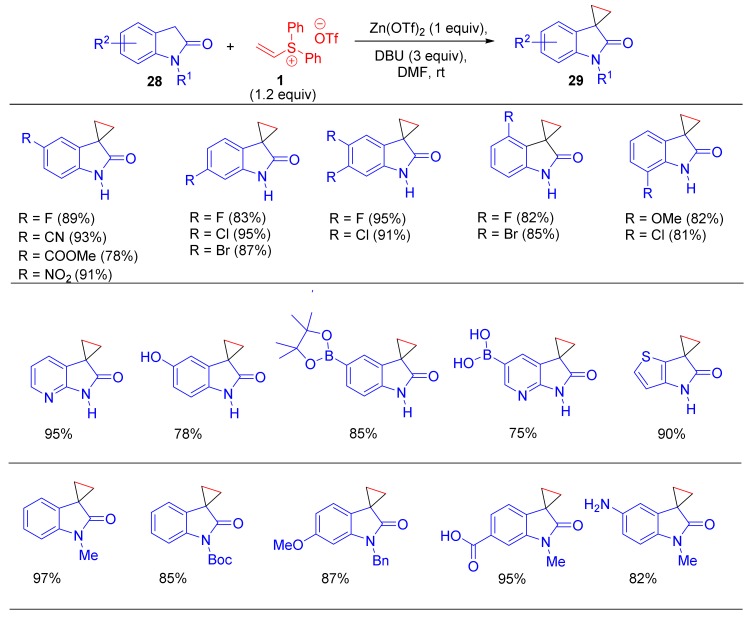
Cyclopropanation of oxindoles with diphenylvinylsulfonium triflate salt.

Recently, Qian and coworkers reported for the first time a zinc triflate-mediated cyclopropanation of both *N*-unsubstituted and *N*-substituted oxindoles **28** with diphenylvinylsulfonium triflate salt to afford **29** (Scheme 7) [36].

The reaction proceeded under ambient conditions and consistently provided very high yields with broad functional group tolerability. A broad reaction scope was observed, with applications employed in the late stage cyclopropanation of complex medicinally-interesting heterocycles (Scheme 7).

**Scheme 8:**
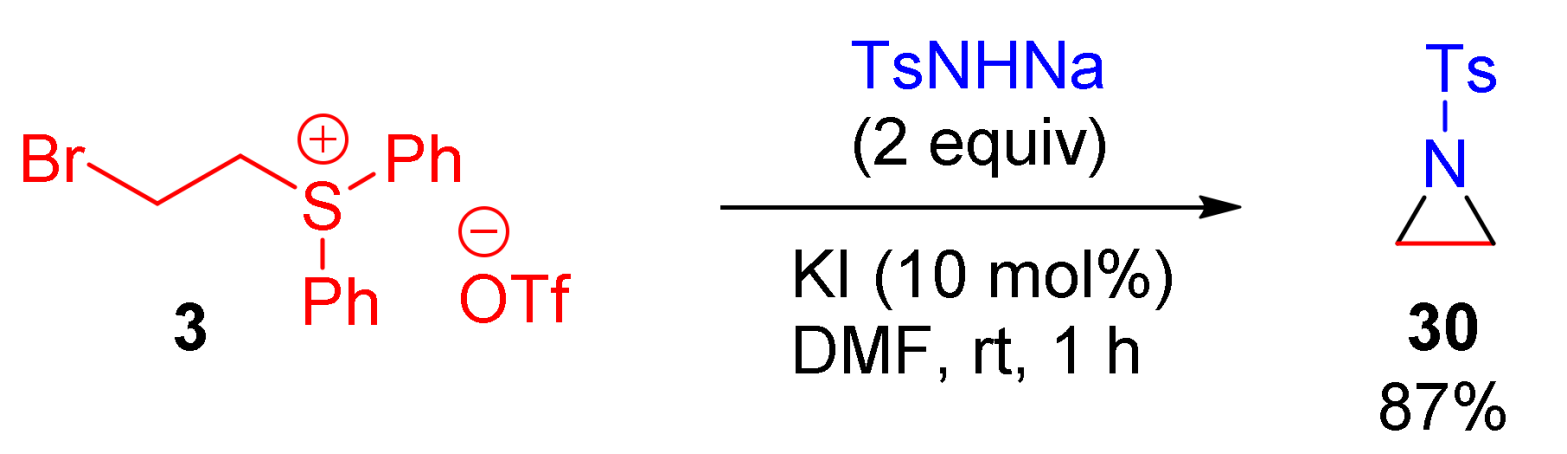
Aziridination using in situ generated vinylsulfonium salts.

In 2004 Mukaiyama introduced a method for the preparation of aziridines from in situ generated vinylsulfonium salts. The vinylsulfonium **1** was generated from the precursor, bromoethyldiphenyl-sulfonium triflate **3**, in the presence of excess nucleophilic base TsNHNa at room temperature, to produce the desired *N*-tosylaziridine **30** in very good yield (Scheme 8) [37].

**Scheme 9:**
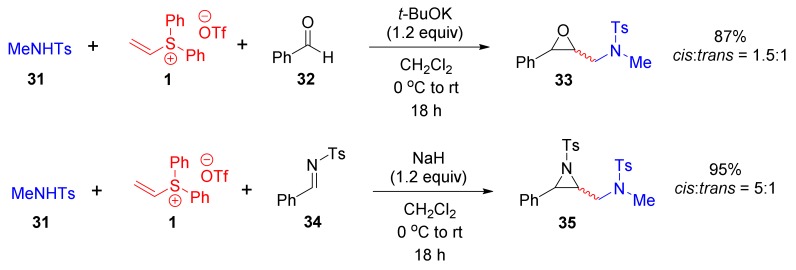
Epoxidation and aziridination from vinylsulfonium salt.

Aggarwal and coworkers reported the three-component coupling reactions of vinylsulfonium salt **1** with a variety of nucleophiles (e.g., **31**) and aldehydes or imines **32** or **34** to provide easy access to epoxides **33** and aziridines **35** respectively (Scheme 9) [30].

### 5.2. Four-Membered Heterocycle Synthesis

**Scheme 10:**
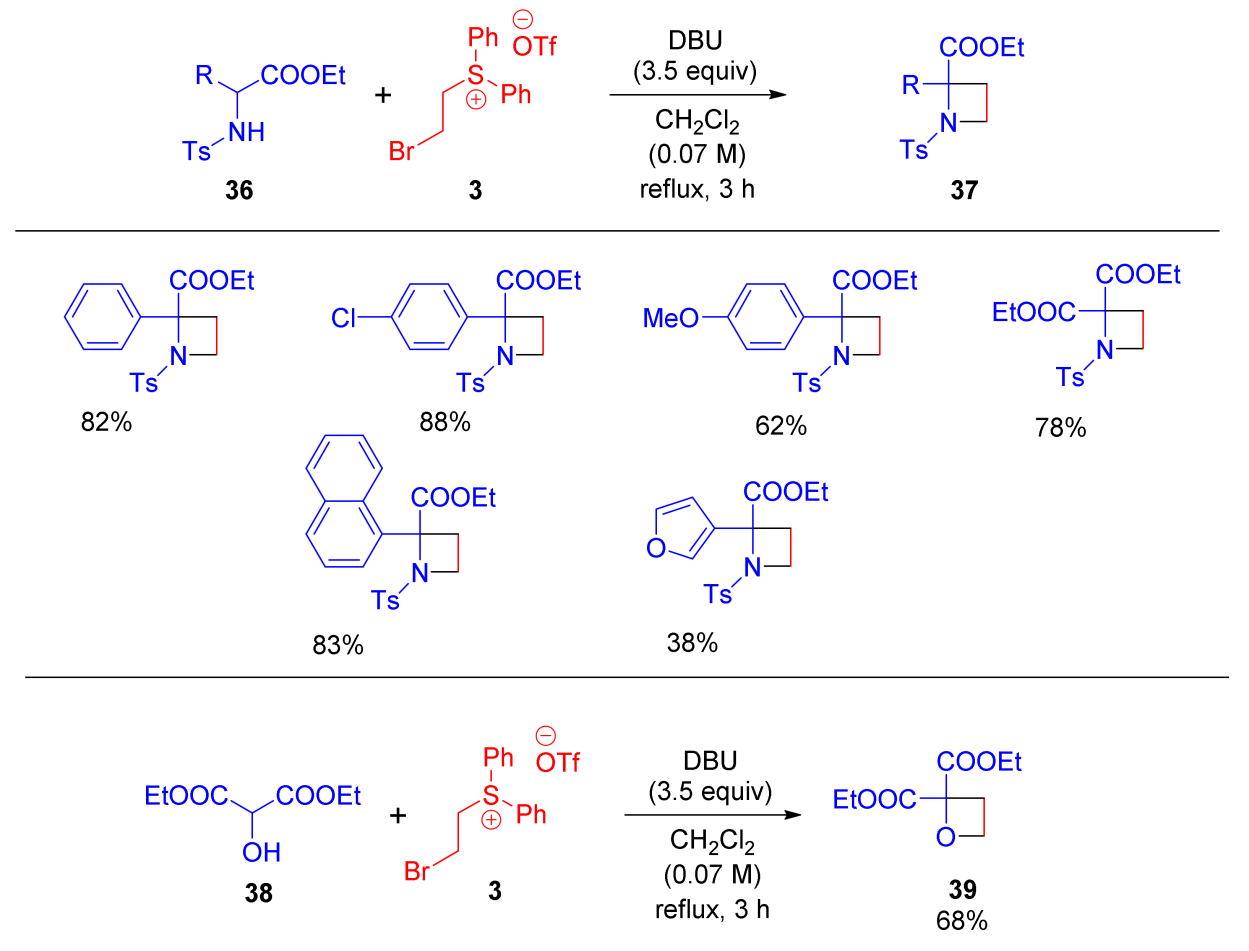
Synthesis of azetidines and oxetanes from in-situ generated vinylsulfonium salt.

Aggarwal and colleagues reported an efficient synthesis of several 2,2-disubstituted azetidines **37** and oxetanes **39** through the reaction of in situ generated vinylsulfonium salt **1**, from the precursor (2-bromoethyl)diphenylsulfonium triflate (**3**), with arylglycine derivatives **36** and hydroxymalonates **38** respectively (Scheme 10) [38]. These reaction procedures are simple, give moderate to high yields, and display broad substrate scope, especially with respect to the arylglycine derivative components.

### 5.3. Five-Membered Heterocycle Synthesis

**Scheme 11:**
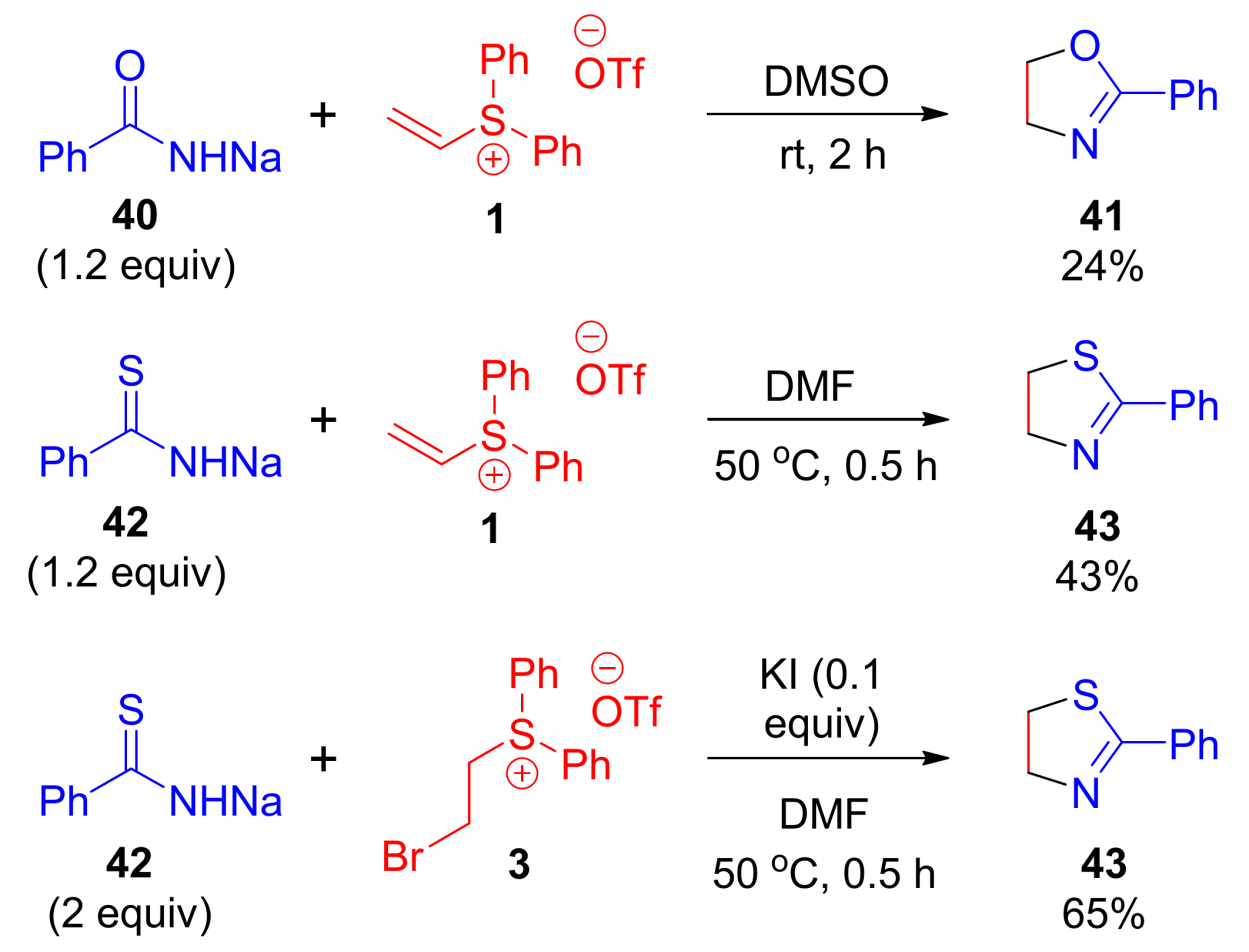
Synthesis of oxazoles and thiooxazoles from vinylsulfonium salt.

The Mukaiyama group reported the synthesis of oxazoles **41** and thiooxazoles **43** with moderate yields by subjecting sodium salts of phenylamide **40** and phenylthioamide **42** to reaction with diphenylvinylsulfonium triflate **1** under mild conditions (Scheme 11) [37].

They were able to improve the yields substantially (up to 65%) by replacing the vinylsulfonium salt **1** with its precursor (2-bromoethyl)diphenylsulfonium triflate **3** and a catalytic amount of potassium iodide (KI is proposed to facilitate conversion of bromide **3** to **1**).

**Scheme 12:**
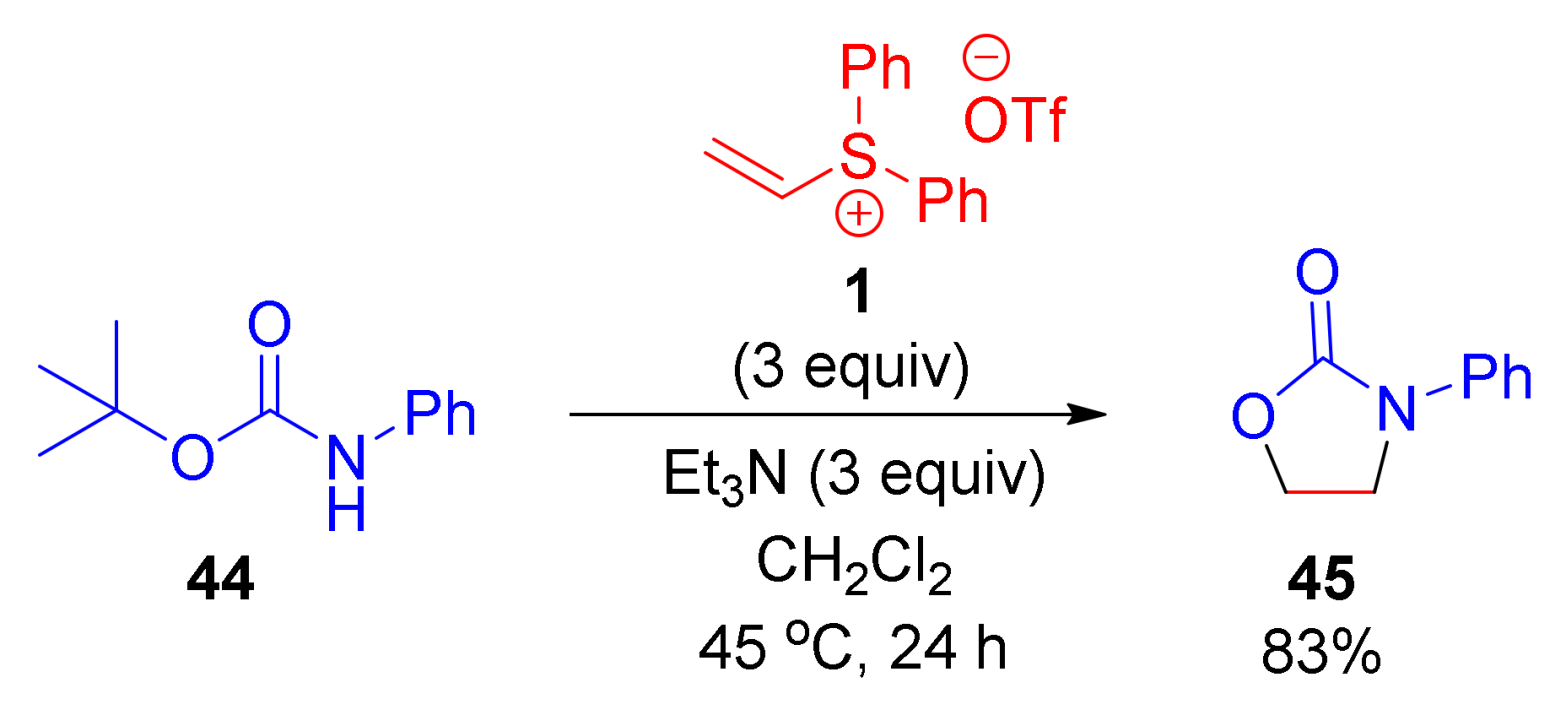
Synthesis of *N*-aryloxazolidin-2-ones from diphenylvinylsulfonium salt.

Xie and coworkers reported an efficient domino reaction of diphenylvinylsulfonium salt **1** with *tert*-butylcarbamates **44** leading to the synthesis of *N*-aryloxazolidin-2-ones **45** [39]. A range of carbamates bearing amide residues with different degrees of substitution were tested and the *N*-aryloxazolidin-2-one products were produced in very high yields (Scheme 12).

**Scheme 13:**
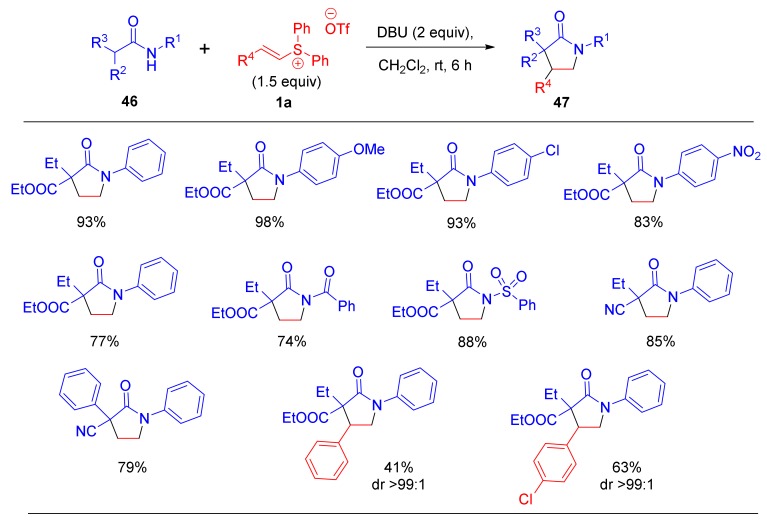
Synthesis of *N*-arylpyrrolidin-2-one from vinylsulfonium salt.

Xie and colleagues during their cyclopropane synthesis, modified the amide reactants by using alkyl substituted *N*-arylmalonylamides **46**, possessing just two acidic protons, and subjected them to reaction with diphenylvinylsulfonium triflates **1a** to produce substituted *N*-arylpyrrolidin-2-ones **47** in moderate to excellent yields (Scheme 13) [33]. This reaction displayed broad substrate scope with respect to both *N*-aryl malonylamide **46** and vinylsulfonium triflates **1a**.

**Scheme 14:**
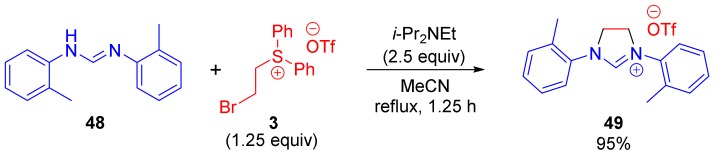
Synthesis of imidazolinium salts from in-situ generated vinylsulfonium salt.

Aggarwal’s group investigated the reaction of vinylsulfonium salt **1**, generated in situ from its precursor (2-bromoethyl)diphenylsulfonium triflate (**3**) in the presence of organic base, with formamidines **48** to synthesize various imidazolinium salts, such as **49**, in high yield, with short reaction times, and in excellent yields (Scheme 14) [40]. The method is applicable to the synthesis of symmetrical and unsymmetrical imidazolinium salts bearing aromatic or aliphatic substituents.

**Scheme 15:**
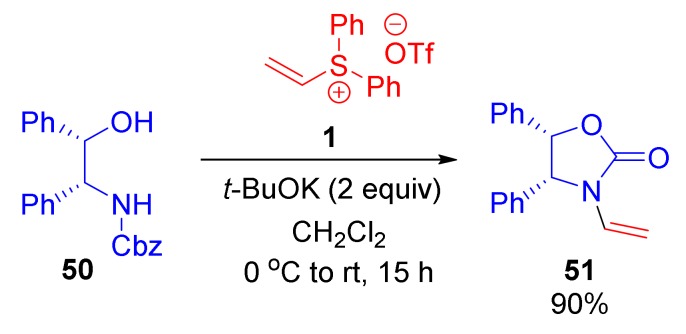
Synthesis of *N*-vinyl oxazolidinones using diphenylvinylsulfonium triflate.

Aggarwal and co-workers also systematically studied the effect of the *N*-protecting group of 1,2-amino alcohols, e.g., **50**, on annulation reactions with vinylsulfonium salt **1** [41]. They observed selective formation of *N*-vinyl oxazolidinones **51** in excellent yield when *N*-Cbz protected 1,2-amino alcohols **50** were treated with vinylsulfonium salt **1** in the presence of inorganic base under mild conditions (Scheme 15).

### 5.4. Six-Membered Heterocycle Synthesis

**Scheme 16:**
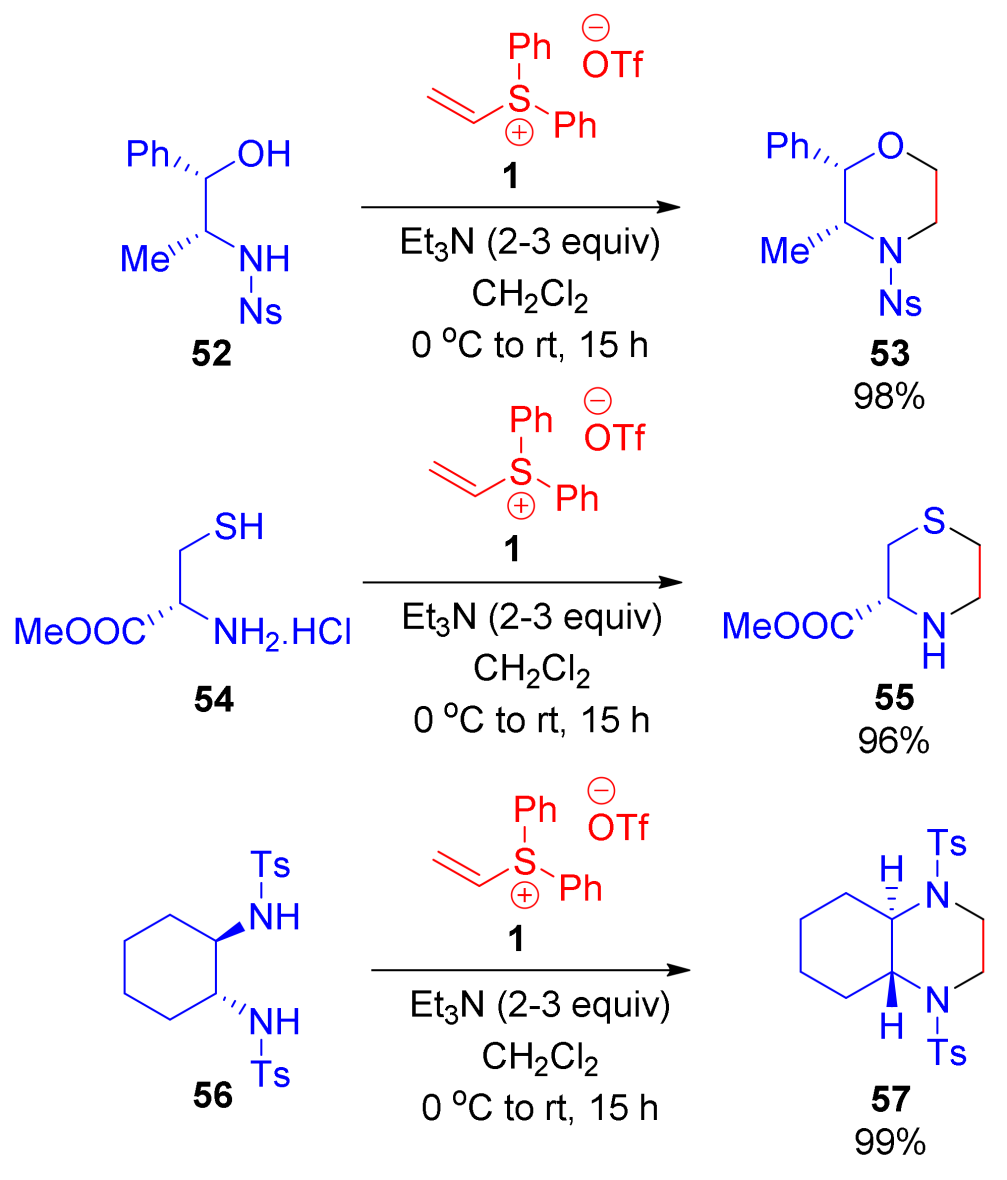
Synthesis of morpholines/thiomorpholines/piperazines using diphenylvinylsulfonium triflate **1**.

In 2008 Aggarwal’s group reported the first [4 + 2] annulation reactions of *β*-heteroatom-substituted amino compounds, e.g., **52**, and diphenylvinylsulfonium triflate **1** for the expedient synthesis of several morpholines (**53**), thiomorpholines (**55**), and piperazines (**57**) (Scheme 16) [42].

Using this procedure, a range of sulfonamide-protected amino alcohols **52**/amino thiols **54**/disulfonamides **56** with different degrees of substitution and stereochemistry gave the corresponding morpholines/thiomorpholines/piperazines in very high yield (morpholines **53**, 94–98%; thiomorpholines **55**, 94–98%; piperazines **57**, 91–99%).

**Scheme 17:**
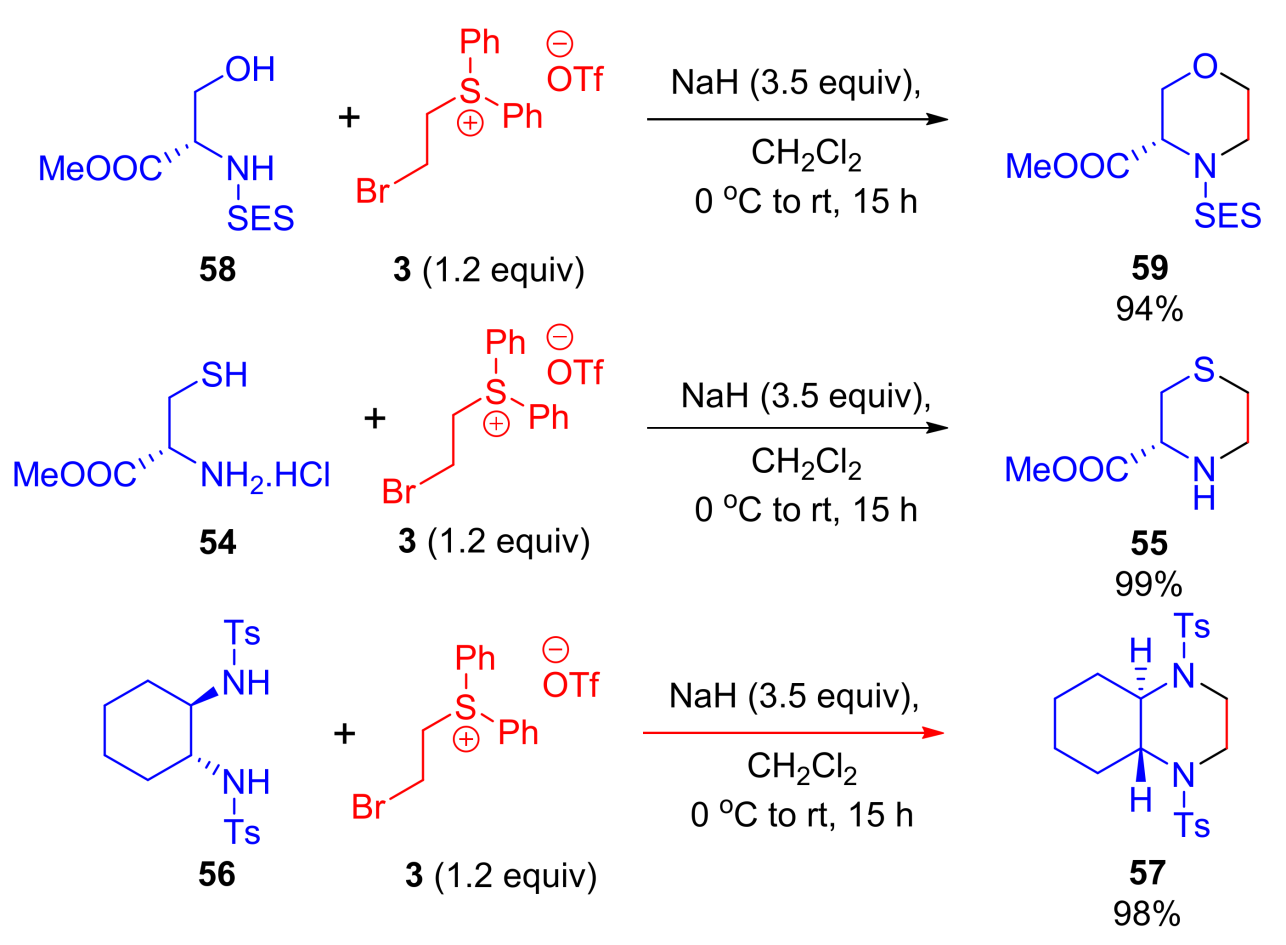
Synthesis of morpholines/thiomorpholines/piperazines using in situ generated diphenylvinylsulfonium triflate.

The same group extended the above annulation methodology using diphenylvinylsulfonium triflate **1**, generated in situ from its precursor (2-bromoethyl)diphenylsulfonium triflate **3**, to undergo reaction with several *β*-amino alcohols/thiols/amines **54**/**56**/**58** and produce an array of morpholines **59**, piperazines **57**, and thiomorpholines **55** [43,44]. Important disubstituted morpholines were synthesized with a range of nitrogen-protecting groups (**59** vs. **57**) in excellent yields (Scheme 17).

**Scheme 18:**
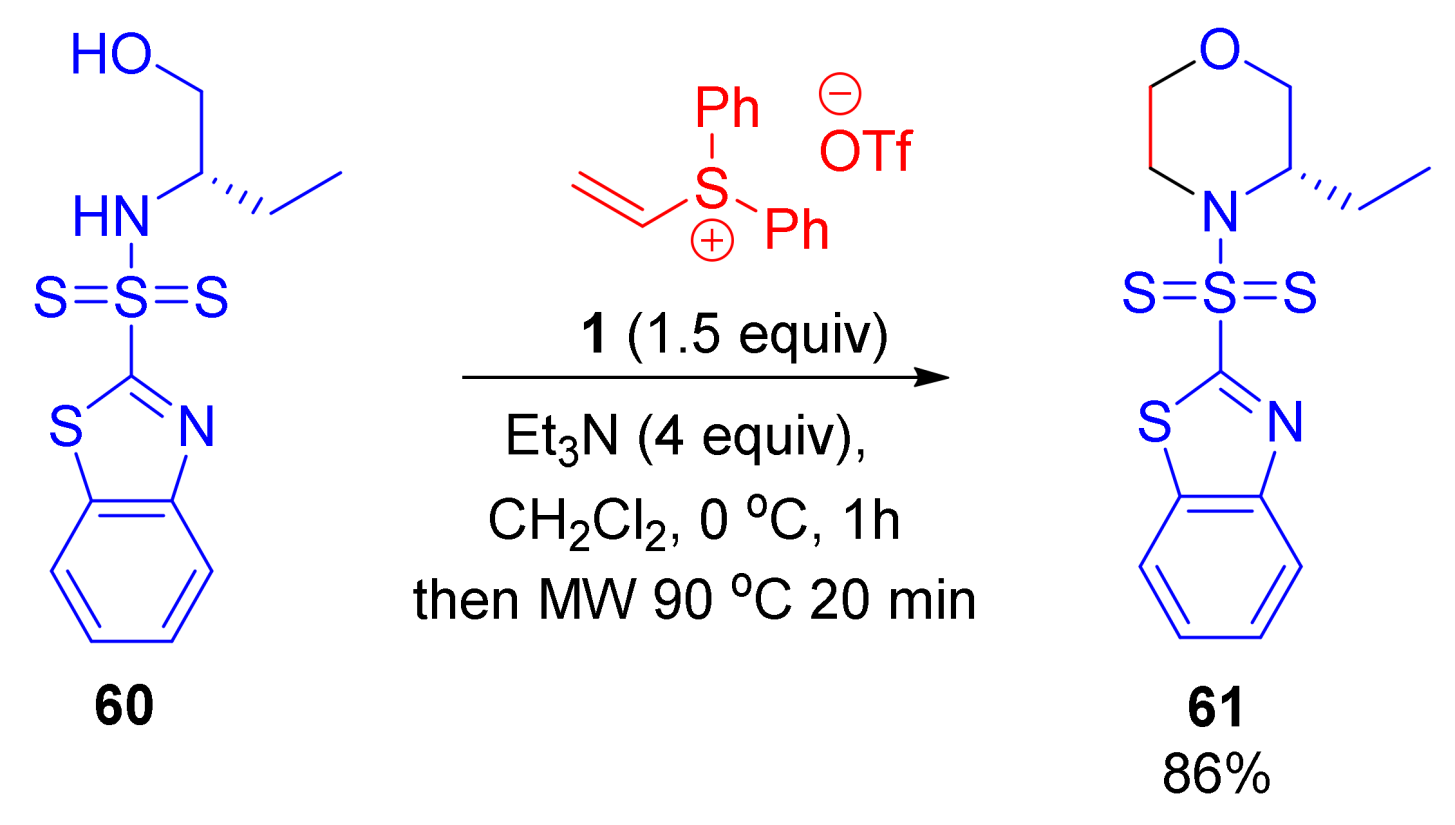
Synthesis of morpholines from 1,2-amino alcohols using diphenylvinylsulfonium triflate.

Kristensen and colleagues reported the microwave-assisted annulation of enantiopure *N*-2-benzo-thiazolesulfonyl (Bts) protected 1,2-amino alcohols, e.g., **60**, with diphenylvinylsulfonium triflate **1** in the presence of triethylamine to furnish Bts-protected morpholines, e.g., **61**, in high yields (Scheme 18) [45].

**Scheme 19:**
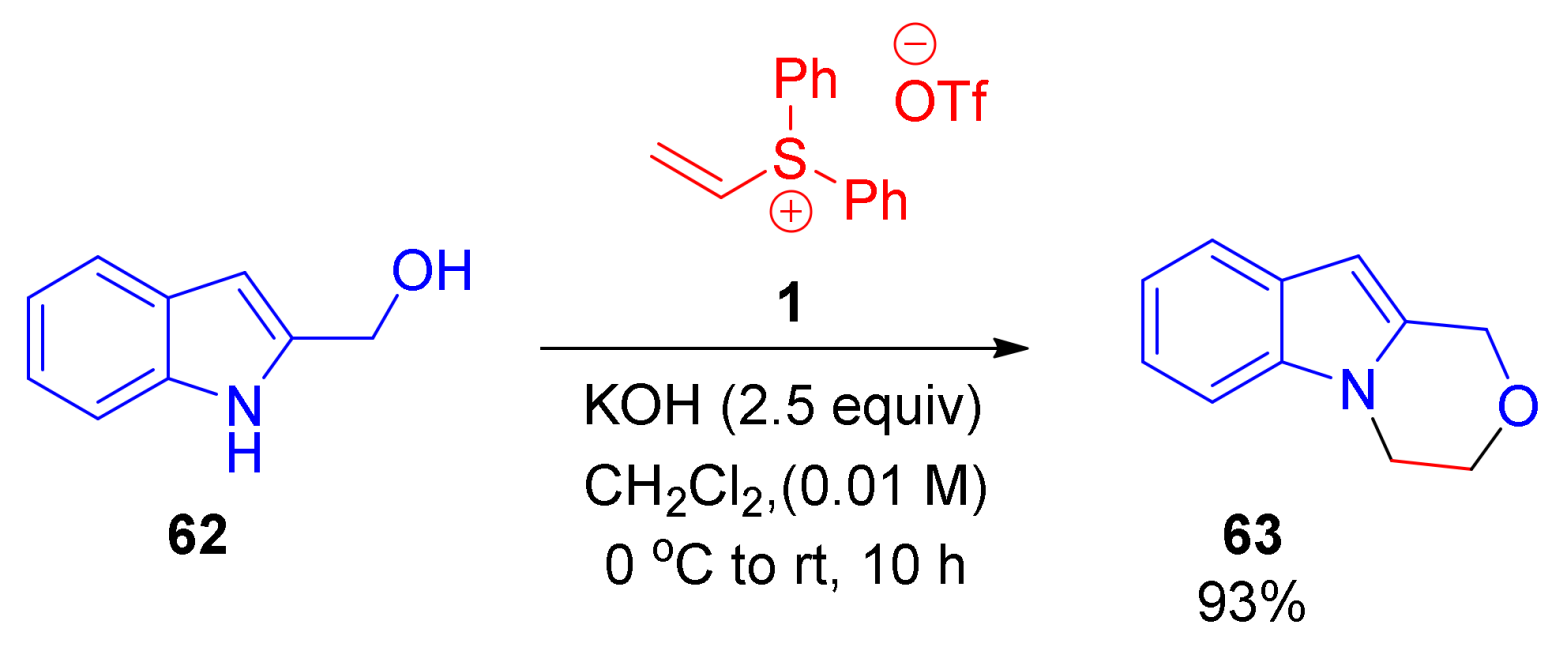
Synthesis of oxazino[4,3-*a*]indole using diphenylvinylsulfonium triflate.

An et al. reported annulation of (1*H*-indol-2-yl)methanols **62** with vinylsulfonium salt **1** for the efficient and general synthesis of oxazino[4,3-*a*]indole architectures **63** in very high yields (Scheme 19) [46].

**Scheme 20:**
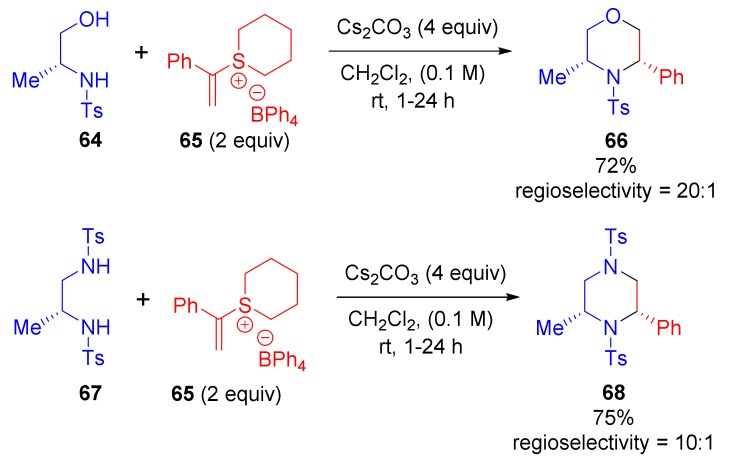
Diastereoselective synthesis of morpholines and piperazines using *α*-phenyl-vinylsulfonium salt.

Aggarwal and colleagues reported the concise synthesis of more complex stereodefined C-substituted morpholines (e.g., **66**) and piperazines (e.g., **68**) in high yields through reaction of 1,2-amino alcohols **64**/disulfonamides **67** with an *α*-phenylvinylsulfonium salt **65** (Scheme 20) [47]. They also achieved excellent regio- and diastereoselectivity by choosing a suitable combination of base and solvent (Scheme 20).

### 5.5. Seven-Membered Heterocycle Synthesis

**Scheme 21:**
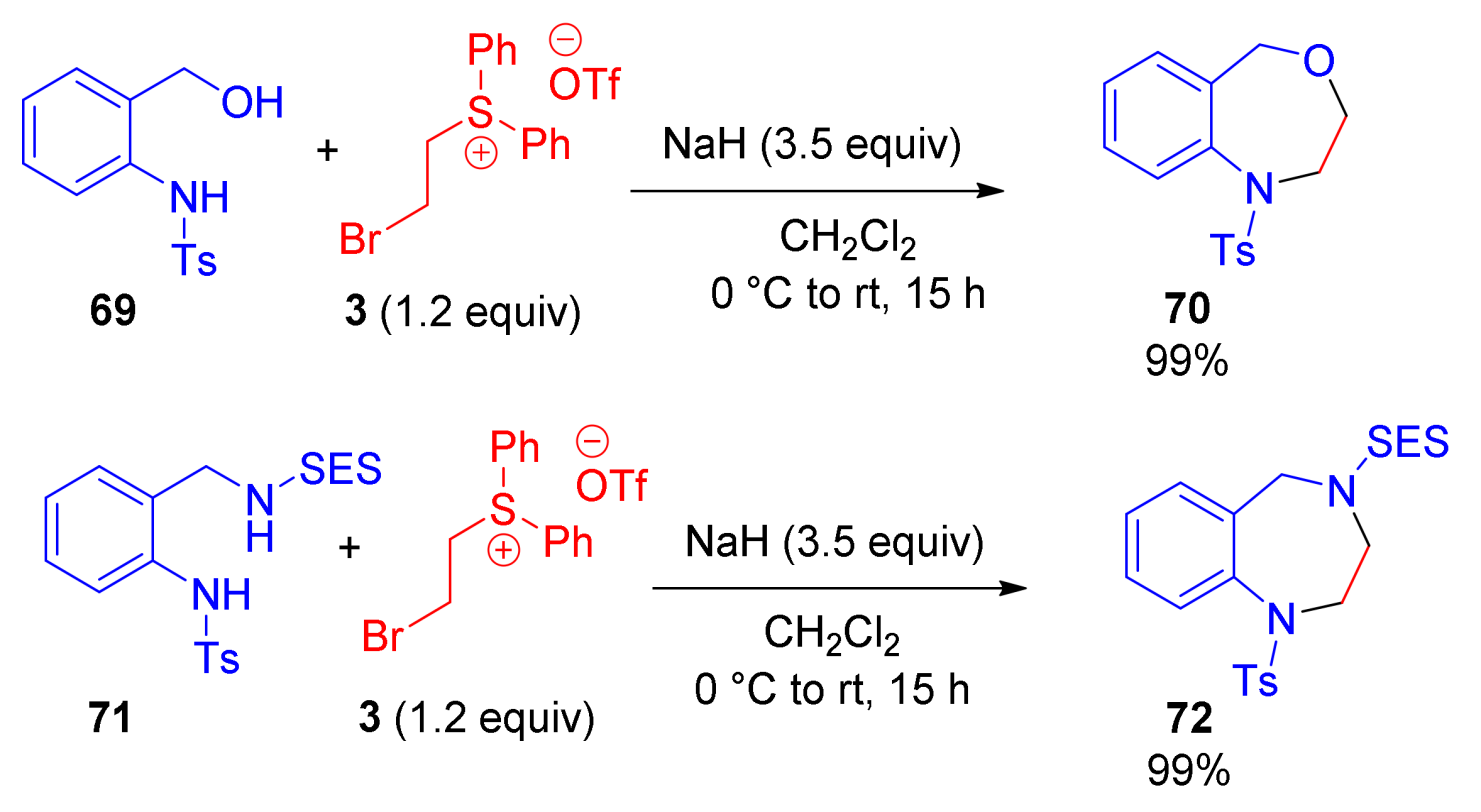
Synthesis of 1,4-oxazepines and 1,4-diazepines using in-situ generated diphenylvinylsulfonium triflate.

Aggarwal and co-workers reported the cyclization reaction of 1,3-aminoalcohols **69**/diamines **71** with vinylsulfonium salt **1**, generated in situ from its precursor (2-bromoethyl)diphenylsulfonium triflate **3** in the presence of base, to produce 1,4-oxazepines **70** and 1,4-diazepines **72** in excellent yields (Scheme 21) [43].

**Scheme 22:**
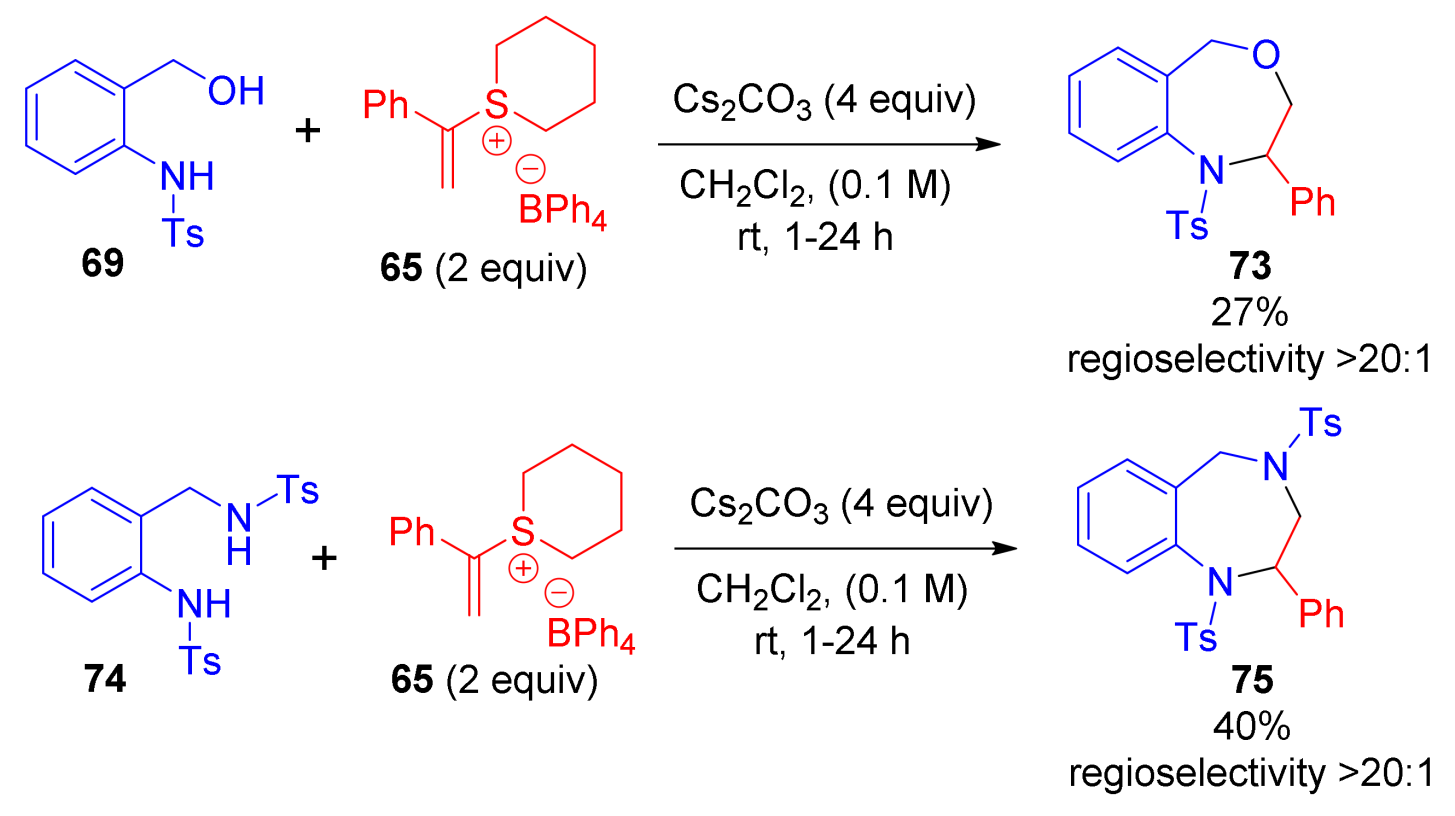
Synthesis of 1,4-oxazepines and 1,4-diazepines using *α*-phenylvinylsulfonium salt.

The same group also extended the prior annulation reaction scope by subjecting *α*-phenylvinylsulfonium salt **65** to reaction with 1,3-aminoalcohols **69**/disulfonamides **74**, thereby producing the corresponding phenyl-substituted 1,4-oxazepines **73** and 1,4-diazepines **75** in moderate yields (Scheme 22) [47].

**Scheme 23:**
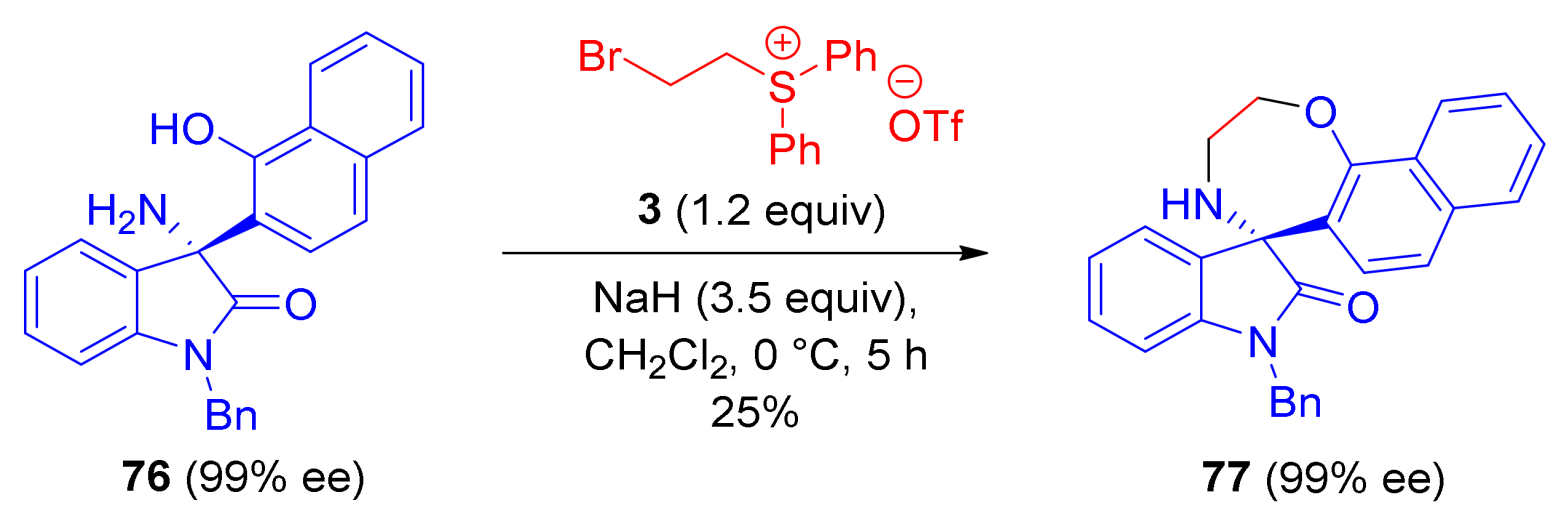
Synthesis of 1,4-naphthoxazepine using in-situ generated diphenylvinylsulfonium salt.

Tanyeli and colleagues recently reported an efficient synthesis of a 1,4-naphthoxazepine derivative **77** using the reaction between enantioenriched 1,3-aminonaphthol **76** and diphenylvinylsulfonium salt **1**, generated in situ from its precursor (2-bromoethyl)diphenylsulfonium triflate **3** in the presence of inorganic base under mild conditions, without any loss in enantiopurity (Scheme 23) [48]. Naphthoxazepines and their derivatives have very high medicinal importance for the central nervous system as antipsychotics, antidepressants, and enzyme inhibitors.

### 5.6. Johnson–Corey–Chaykovsky-Type Reactions for Fused Heterocycle Synthesis

**Scheme 24:**
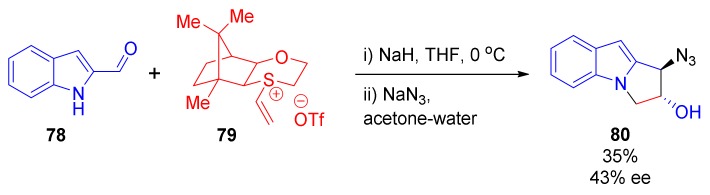
Enantioselective synthesis of tricyclic azido alcohol using chiral vinylsulfonium salt.

Jimenez and co-workers introduced a camphor-derived vinylsulfonium salt **79** as a reagent for enantioselective cycloannulation reactions [49]. This chiral vinylsulfonium salt **79** underwent reaction with indole-2-carboxaldehyde **78** to afford an oxirane intermediate which upon ring opening with azide produced tricyclic azidoalcohol **80** in moderate yield and in moderate enantiomeric excess (Scheme 24).

**Scheme 25:**
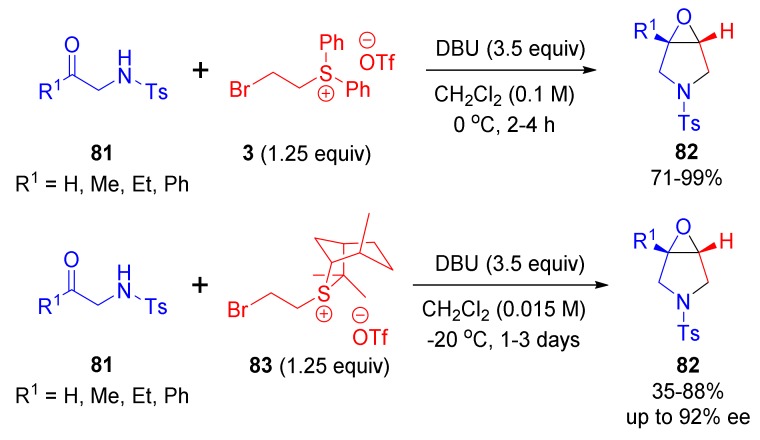
Synthesis of bicyclic epoxide using in-situ generated vinylsulfonium salt.

Along similar lines, the synthesis of fused bicyclic epoxide heterocycles **82** was reported by Aggarwal and co-workers, whereby tandem Michael addition/Johnson–Corey–Chaykovsky-type reactions were employed [50]. The reaction of vinylsulfonium salt **1**, generated in situ from its precursor (2-bromoethyl)diphenylsulfonium triflate **3** in the presence of base (DBU) under mild conditions, with *α*-amino ketones **81** afforded bicyclic epoxides **82** in good to excellent yields. When a chiral (2-bromoethyl)sulfonium reagent **83** was employed, the desired bicyclic epoxides were obtained in moderate to excellent yields and with very good enantioselectivities observed (Scheme 25).

**Scheme 26:**
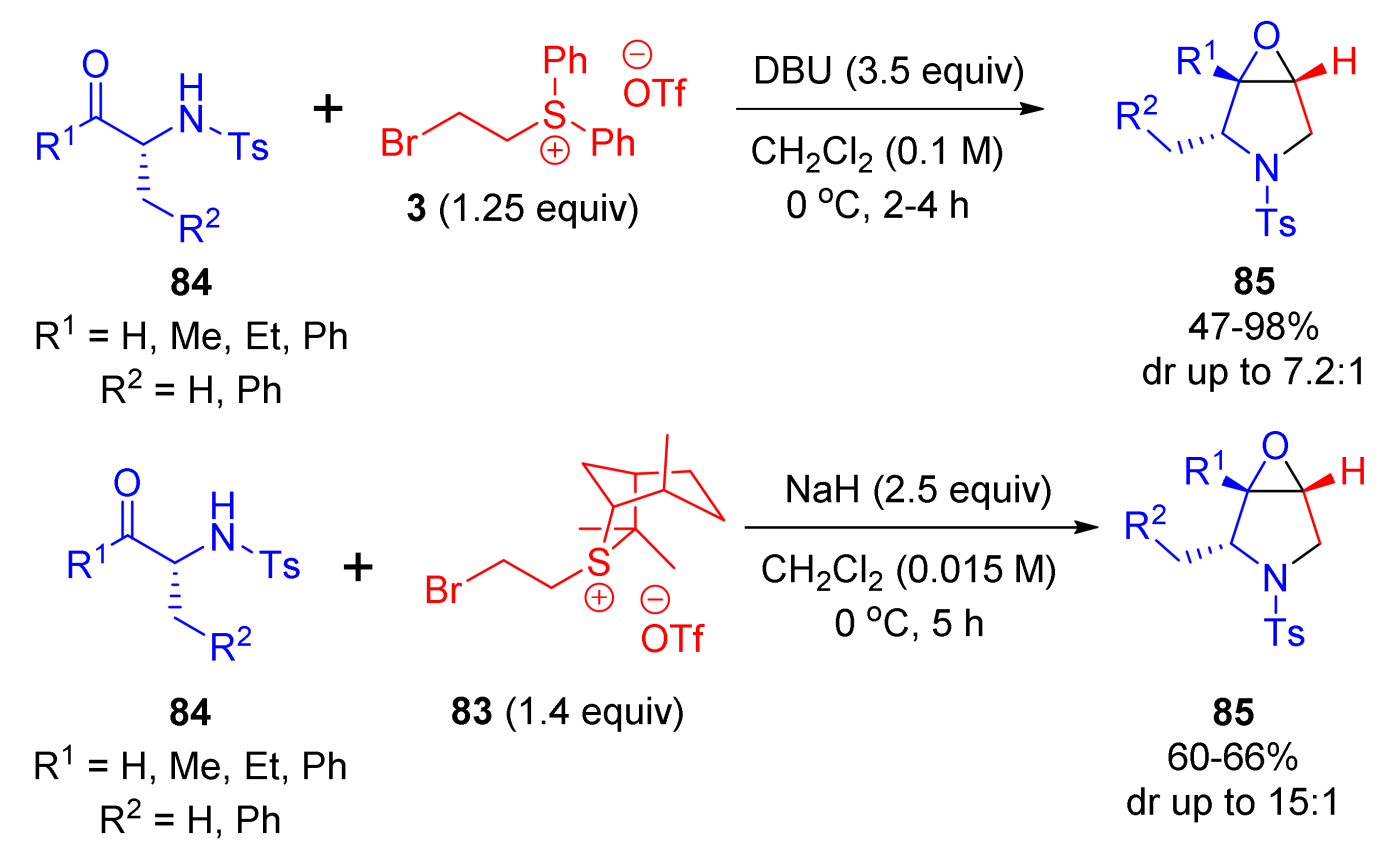
Diastereoselective synthesis of bicyclic epoxides using in-situ generated vinylsulfonium salt.

A diastereoselective variant of this reaction was also reported, starting from enantiopure *α*-substituted *α*-amino ketones **84** [50]. Employing the achiral (2-bromoethyl)diphenylsulfonium triflate **3**, good to excellent yields and good levels of diastereoselectivity were observed for the synthesis of the fused bicyclic epoxide *N*-heterocycle **85**. In addition, enhanced levels of diastereocontrol were observed when using the chiral bromoethylsulfonium triflate **83** (Scheme 26).

**Scheme 27:**
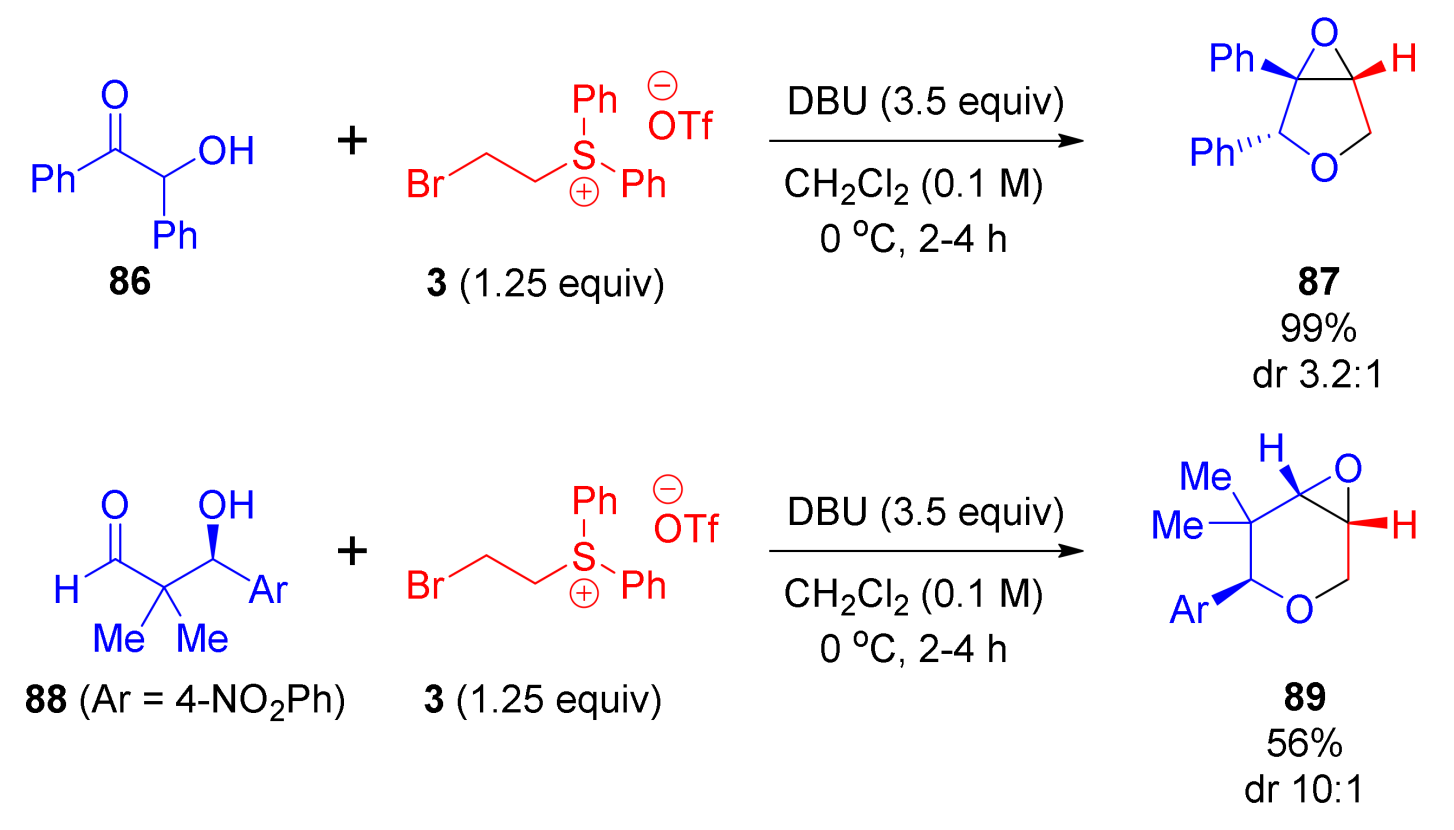
Diastereoselective synthesis of bicyclic epoxides using in-situ generated vinylsulfonium salt.

*α*-Hydroxyketone **86** underwent epoxy annulation reactions with vinylsulfonium salt **1**, generated in situ from (2-bromoethyl)diphenylsulfonium triflate **3** with base, to produce bicyclic epoxide **87** in excellent yield but with moderate diastereoselectivity. On the other hand, *β*-hydroxy aldehyde **88** underwent the same epoxy annulation reaction in good yield and with good diastereoselectivity (Scheme 27) [50].

**Scheme 28:**
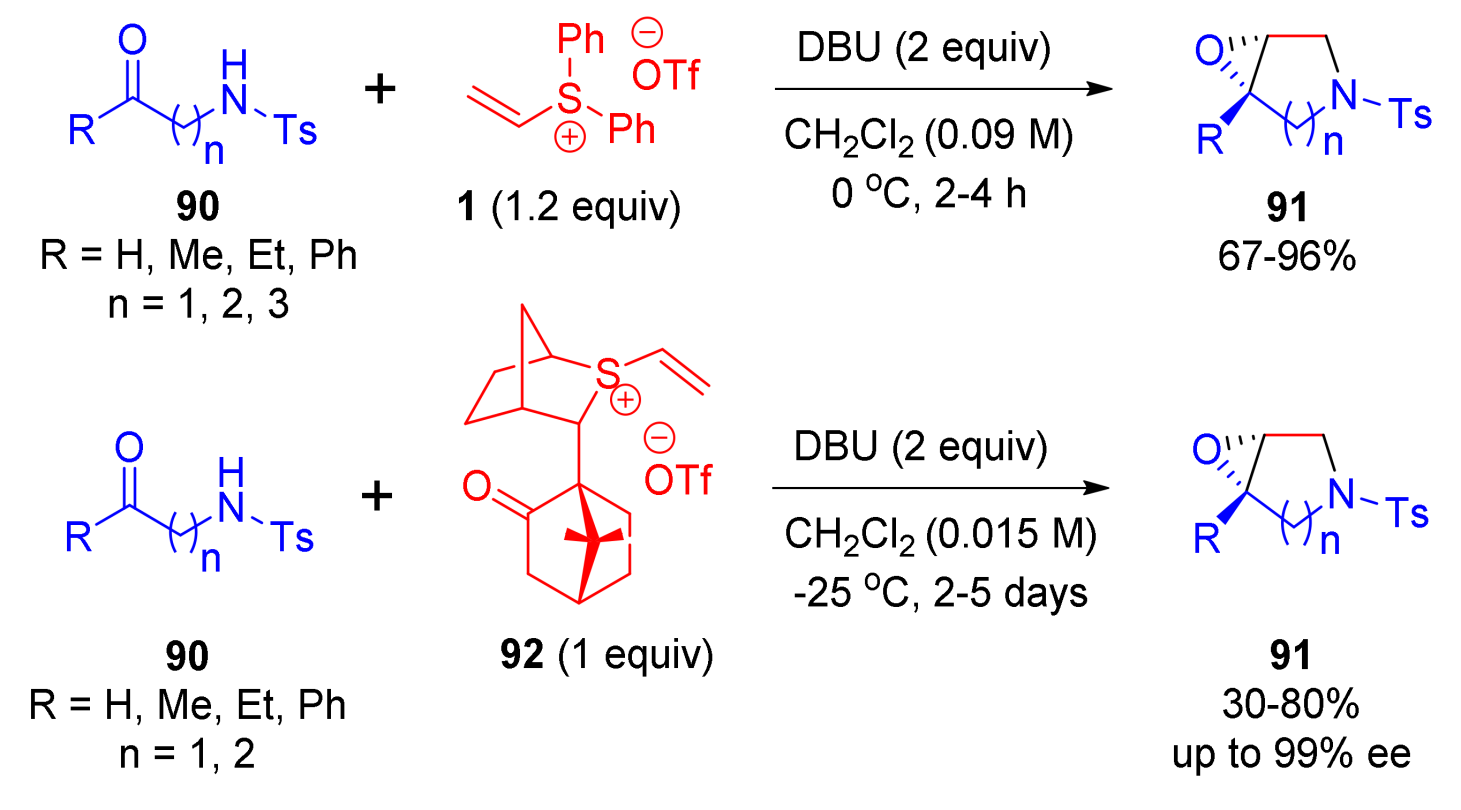
Synthesis of bicyclic epoxides using vinylsulfonium salts.

Aggarwal and co-workers also developed a new epoxy-annulation method for converting *α*-, *β*-, and *γ*-amino aldehydes and -ketones **90** into racemic five-, six-, and seven-membered epoxide-fused heterocycles **91** through reaction with diphenylvinylsulfonium triflate **1** under mild reaction conditions [31]. An asymmetric variant for the synthesis of five- and six-membered heterocycles was developed through the use of chiral vinylsulfonium salt **92** and excellent levels of asymmetric induction were achieved (Scheme 28).

**Scheme 29:**
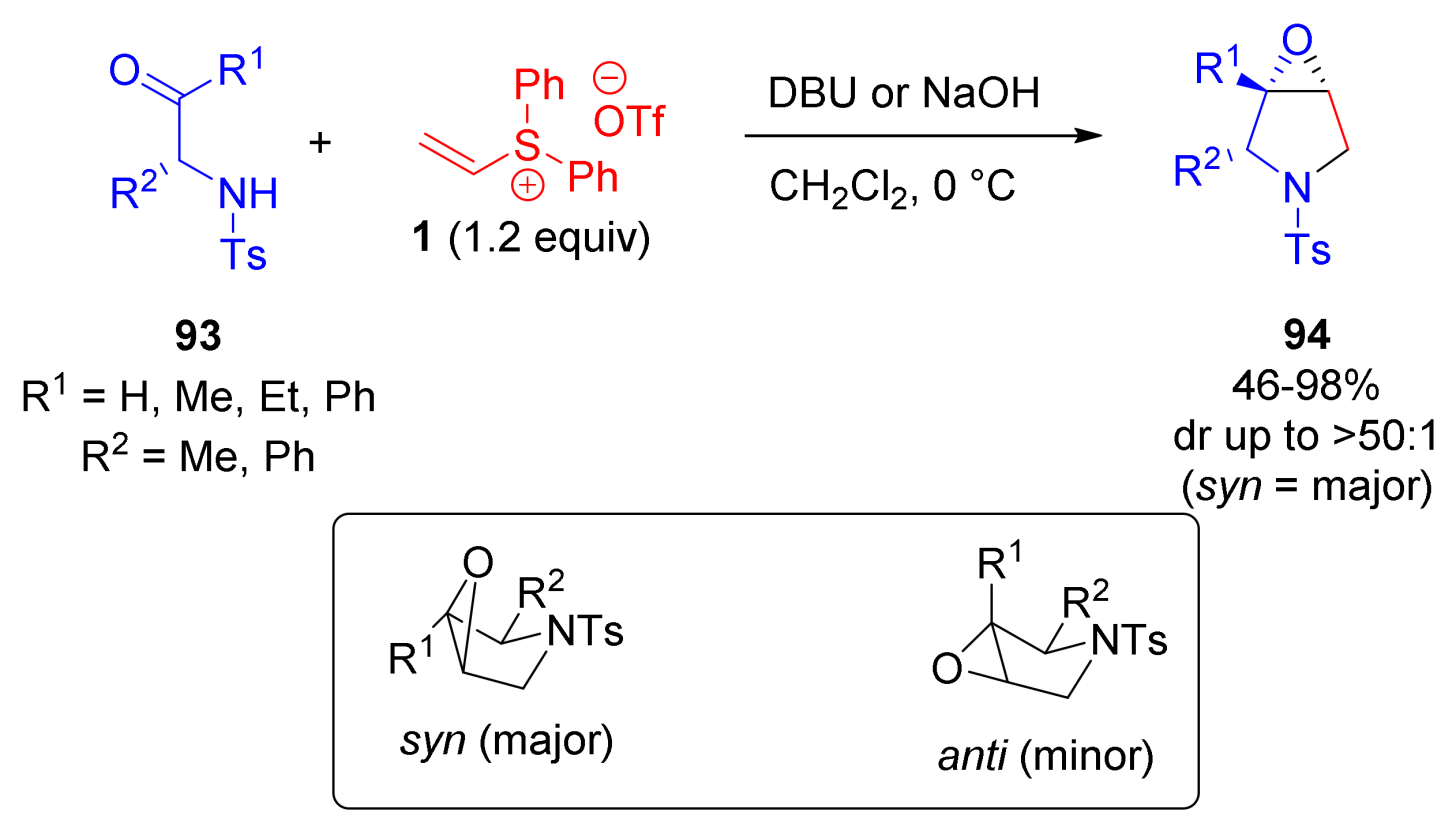
Diastereoselective synthesis of bicyclic epoxide using vinylsulfonium salt.

The same group also reported diastereoselective diphenylvinylsulfonium salt **1**-mediated epoxy annulation reactions with enantiopure *α*-amino ketone **93** giving *syn*-bicyclic epoxides **94** predominantly [51]. The observed diastereoselectivity of these epoxy annulation reactions depended upon the steric properties of the ketones as well as that of the vinylsulfonium salt employed. With the larger phenyl ketone **93** (R^1^ = Ph), highest diastereoselectivity (>50:1) was achieved with both achiral and chiral vinylsulfonium salts, while the intermediate-size dialkylketones (R^1^ = Et or Me) gave moderate levels of diastereoselectivity in the presence of the achiral vinylsulfonium salt. However, intermediate-size dialkylketones underwent reaction with chiral vinylsulfonium salt with excellent diastereoselectivity. The reaction of aldehydes with achiral vinylsulfonium salt **1** displayed almost no stereocontrol (Scheme 29).

**Scheme 30:**
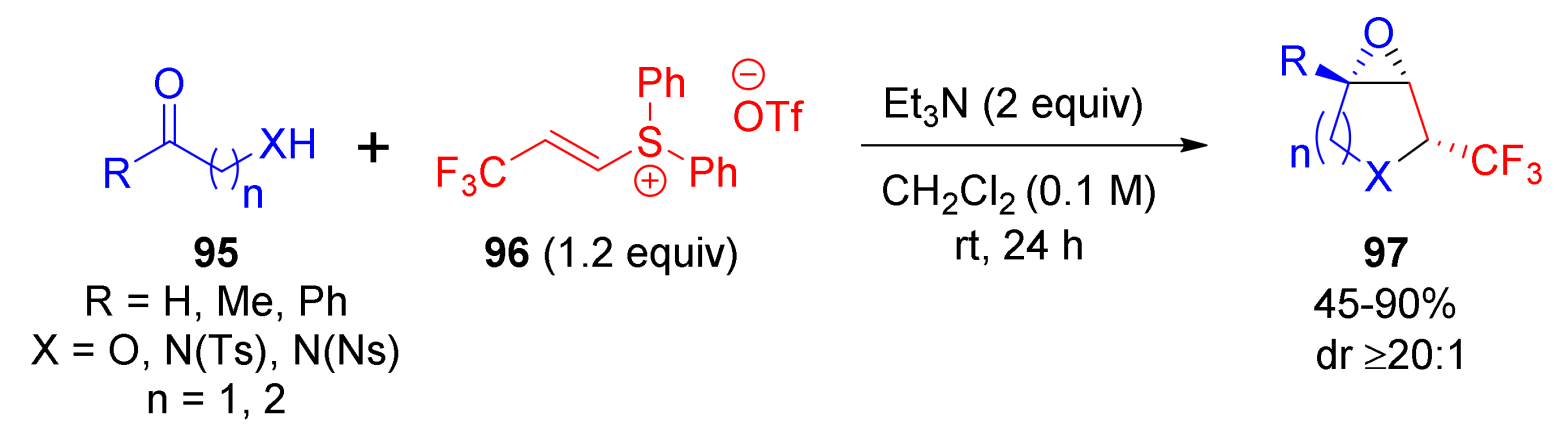
Synthesis of bicyclic epoxide heterocycles using *β*-trifluoromethyl-substituted diphenylvinylsulfonium triflate.

*β*-Trifluoromethyl-substituted diphenylvinylsulfonium triflate **96** was introduced by Aggarwal and colleagues for epoxy annulation reactions with *α*- or *β*-amino ketones/hydroxyketones **95** to produce *α*-trifluoromethyl-substituted bicyclic expoxy-fused heterocycles **97** (pyrrolidines, piperidines and tetrahydrofurans) in high yield and with high diastereoselectivity (Scheme 30) [52].

**Scheme 31:**
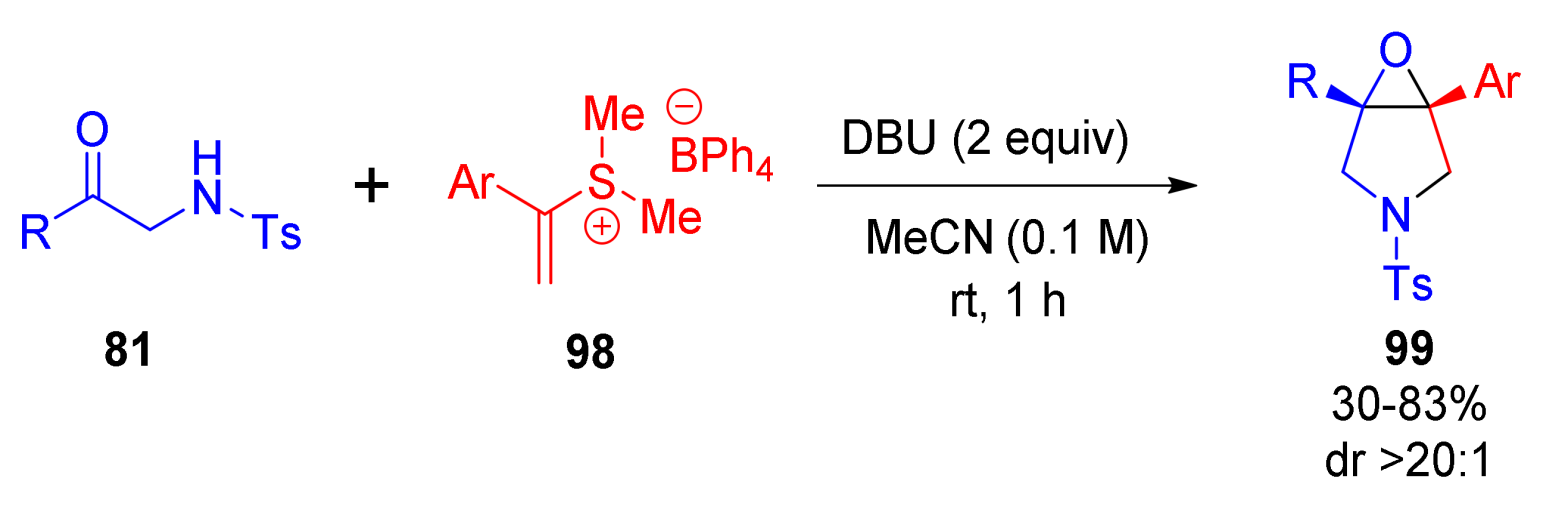
Synthesis of bicyclic epoxide heterocycles using *α*-substituted vinylsulfonium salt.

Aggarwal and co-workers later introduced *α*-substituted vinylsulfonium tetraphenylborates **98** as stable isolable salts [53]. Epoxy annulation reactions of the *α*-substituted vinylsulfonium tetraphenylborates **98** with *α*-amino ketones **81** gave bicyclic expoxy-fused heterocycles **99** in good yields and with high diastereoselectivity (Scheme 31).

**Scheme 32:**
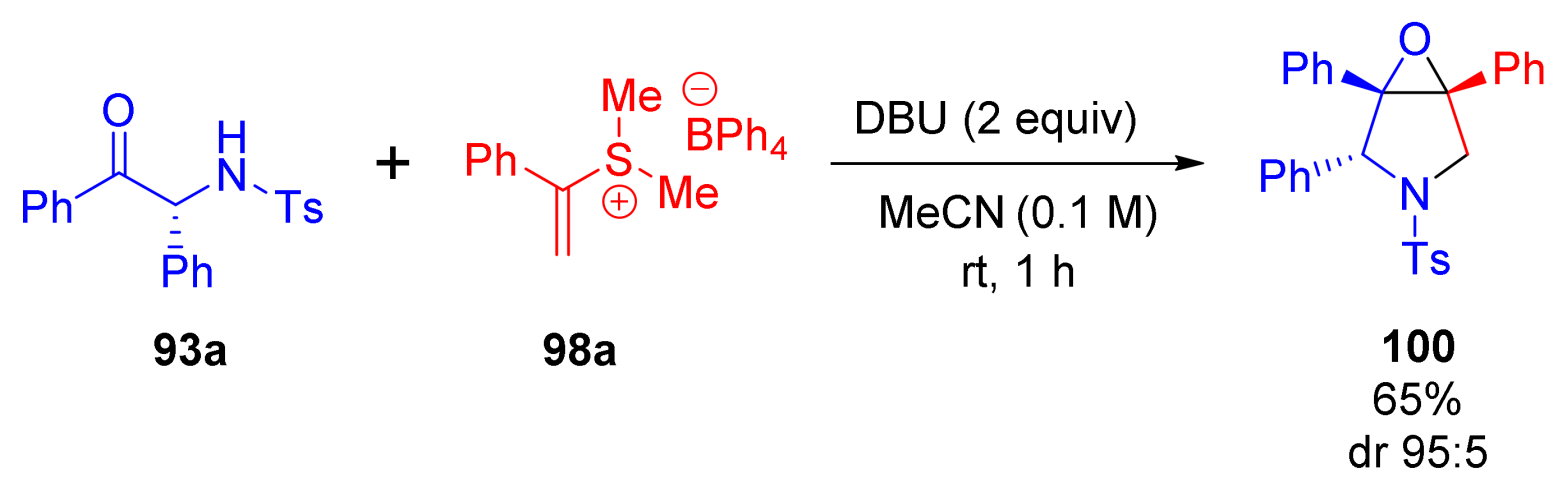
Diastereoselective synthesis of bicyclic epoxide using *α*-substituted vinylsulfonium salt.

The same reaction starting from an enantioenriched *α*-amino ketone **93a** gave a bicyclic epoxy-fused heterocycle **100** with high yield and high diastereoselectivity (Scheme 32) [53].

**Scheme 33:**
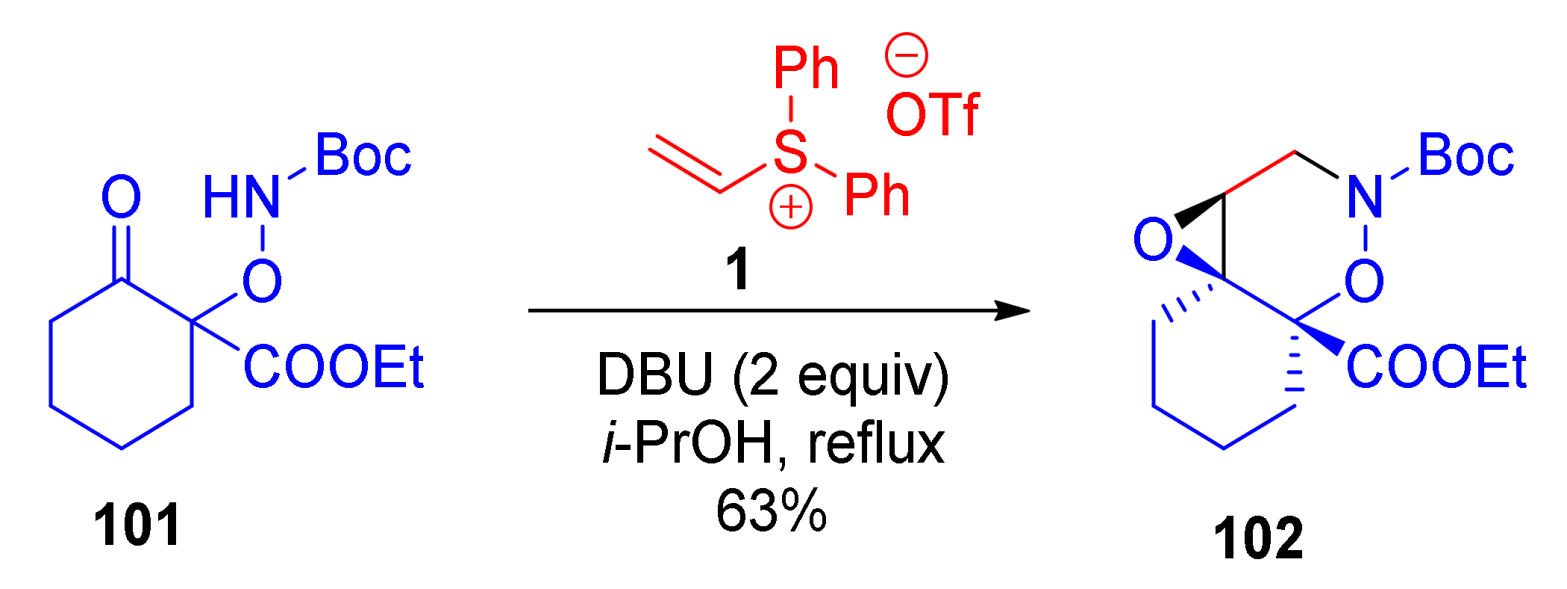
Synthesis of epoxy-oxazine using diphenylvinylsulfonium salt.

Frazier et al. reported an excellent diastereoselective annulation reaction of *α*-aminooxy-carbonyl compound **101** with diphenylvinylsulfonium triflate **1** in the presence of organic base (DBU) to furnish a highly substituted epoxy-oxazine **102** as a single diastereomer in good yield (Scheme 33) [54].

**Scheme 34:**
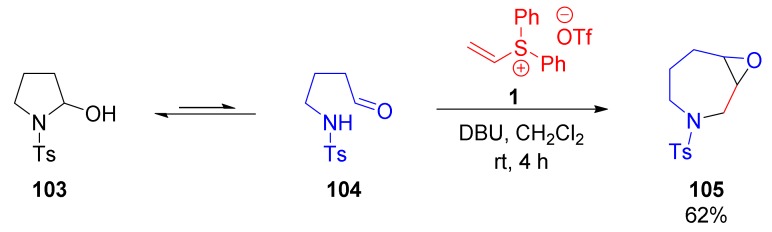
Synthesis of epoxy azepines using diphenylvinylsulfonium salt.

The *γ*-amino-aldehyde **104** underwent epoxy annulation reactions with diphenylvinylsulfonium triflate **1** to access challenging seven-membered heterocyclic fused epoxides **105** in good yield (Scheme 34) [31]. The *γ*-amino-aldehyde **104** exists predominantly in the ring-closed hemiaminal form **103**, which leads to a low concentration of the required nucleophile in the reaction mixture, and ring closure to form the seven-membered ring is much more difficult to accomplish due to the large ring size. However, after optimization this substrate provided a new route to functionalized bicyclic epoxy azepines **105** (Scheme 34).

**Scheme 35:**
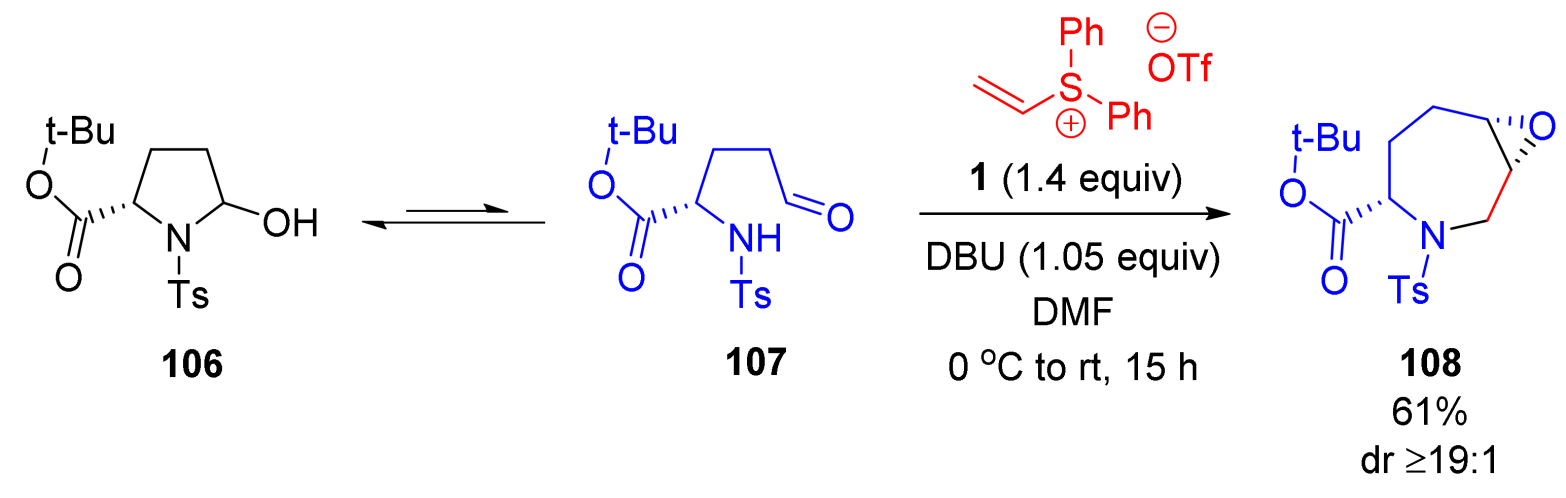
Diastereoselective synthesis of epoxy azepines using diphenylvinylsulfonium salt.

Later, the same group further extended this reaction to the diastereoselective synthesis of perhydroazepines **108** (Scheme 35) [29]. Starting from chiral 5-substituted hemiaminal **106**, epoxy annulation reactions with diphenylvinylsulfonium triflate **1** gave *cis*-epoxides **108** in good yield and with good to excellent diastereoselectivity. The steric size and position of the substituents were found to play an important role in controlling diastereoselectivity.

**Scheme 36:**
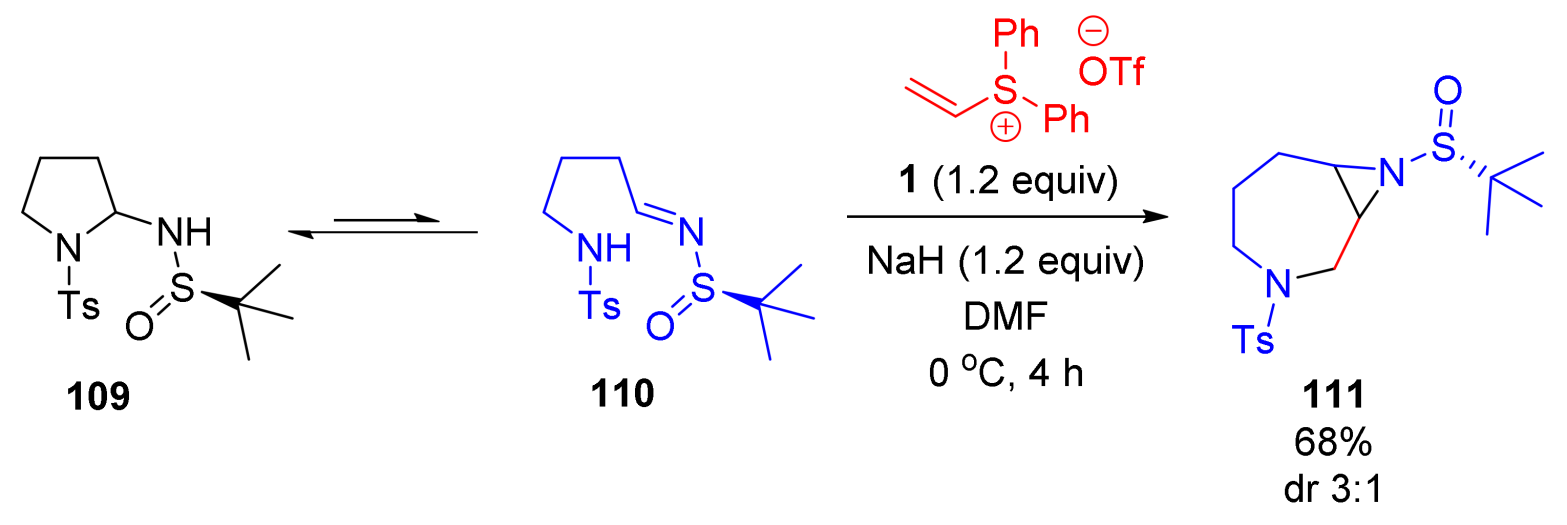
Synthesis of hexahydroazepine using diphenylvinylsulfonium salt.

The same idea was extended to the synthesis of bicyclic aziridine fused-heterocycles **111 [31]**. Aziridine-annulation reaction of chiral aminal **109** with diphenylvinylsulfonium salt **1** furnished the desired hexahydroazepine **111** in good yield and with moderate diastereoselectivity (Scheme 36).

**Scheme 37:**
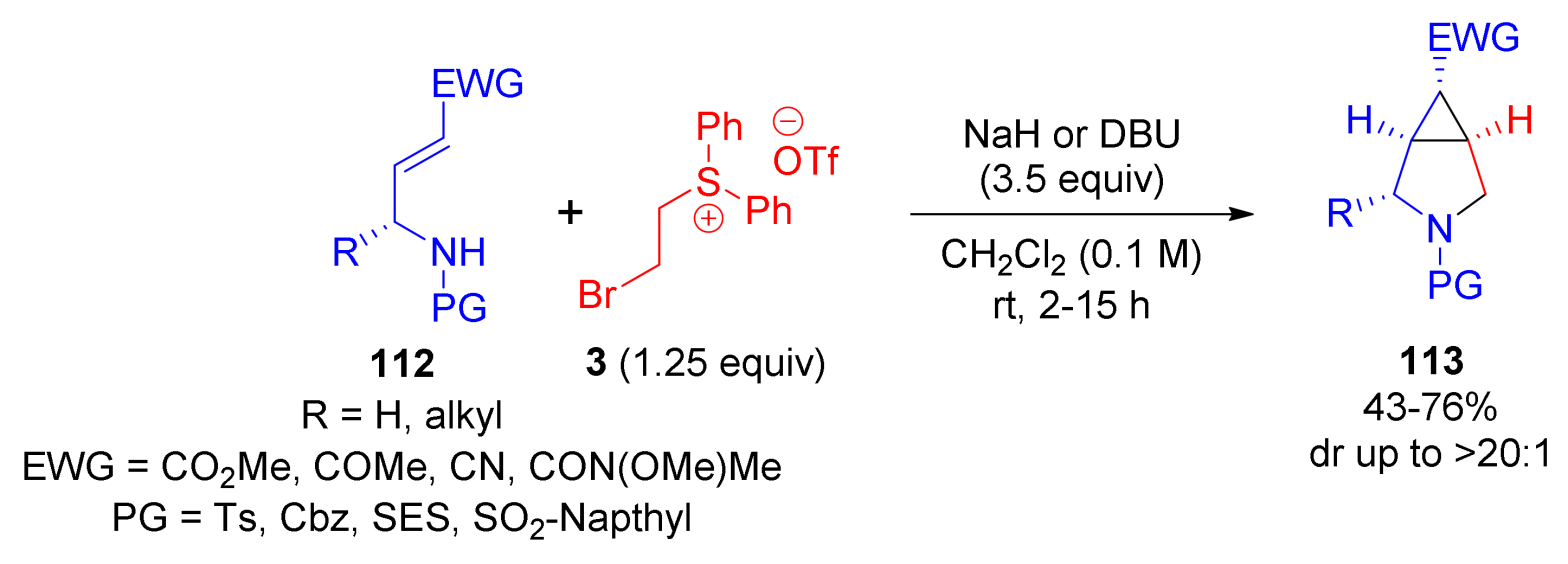
Synthesis of bicyclic cyclopropane-fused heterocycles using in situ generated vinylsulfonium salt.

Aggarwal and colleagues reported an efficient and versatile method for the formation of bicyclic cyclopropane-fused heterocycles **113** by subjecting *β*,*γ*-unsaturated amine derivatives **112** to reaction with diphenylvinylsulfonium triflate **1**, generated in situ from the precursor (2-bromoethyl)diphenylsulfonium triflate **3** [55]. Allylic amine derivatives **112** bearing Michael acceptors with various electron-withdrawing groups were successfully converted to the desired bicyclic 3-azabicyclo[3.1.0]hexanes **113** in moderate to good yields and with moderate to excellent diastereoselectivity (Scheme 37).

**Scheme 38:**
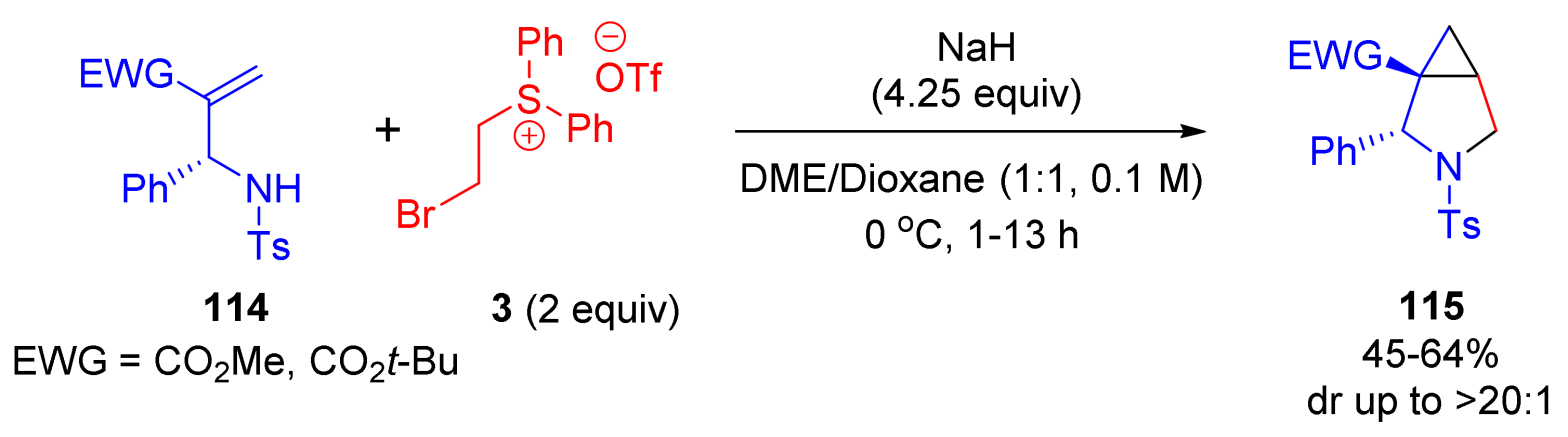
Diastereoselective synthesis of bicyclic cyclopropane-fused heterocycles using in-situ generated vinylsulfonium salt.

This methodology was further explored by using chiral aza-Morita–Baylis–Hillman adducts **114** as reactants [55]. The annulation reaction with in situ generated diphenylvinylsulfonium triflate **1** gave the desired products **115** in moderate to good yields, and with excellent diastereoselectivity (Scheme 38).

**Scheme 39:**
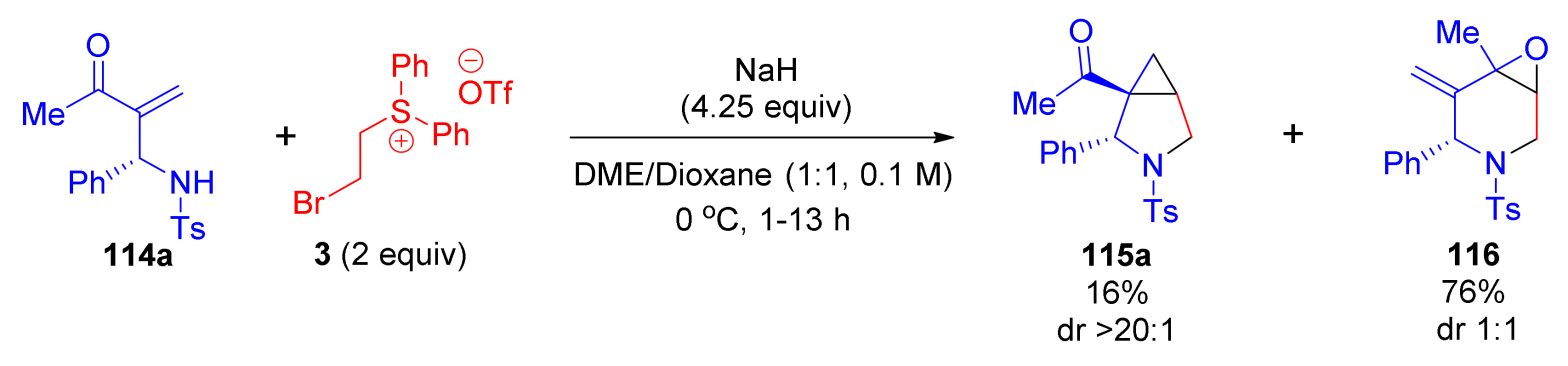
Diastereoselective synthesis of bicyclic cyclopropane-fused heterocycles using in situ generated vinylsulfonium salt.

While the reaction of aza-Morita–Baylis–Hillman adducts **114** possessing an ester group as electron withdrawing group worked very well, unsaturated ketone **114a** was less effective, giving the product **115a** in low yield, albeit with excellent diastereoselectivity. In this case, a competing epoxy-annulation reaction, resulting from 1,2-addition of the sulfur ylide intermediate onto the methyl ketone (6-*exo*-trig cyclization to give **116**) dominated, instead of the desired 1,4-addition onto the Michael acceptor (6-*endo*-trig cyclization to give **115a**) (Scheme 39) [55].

## 6. Reactions of Vinylsulfoxonium Salts

### 6.1. Asymmetric Synthesis of Anti-Homopropargylic Alcohols 

**Scheme 40:**
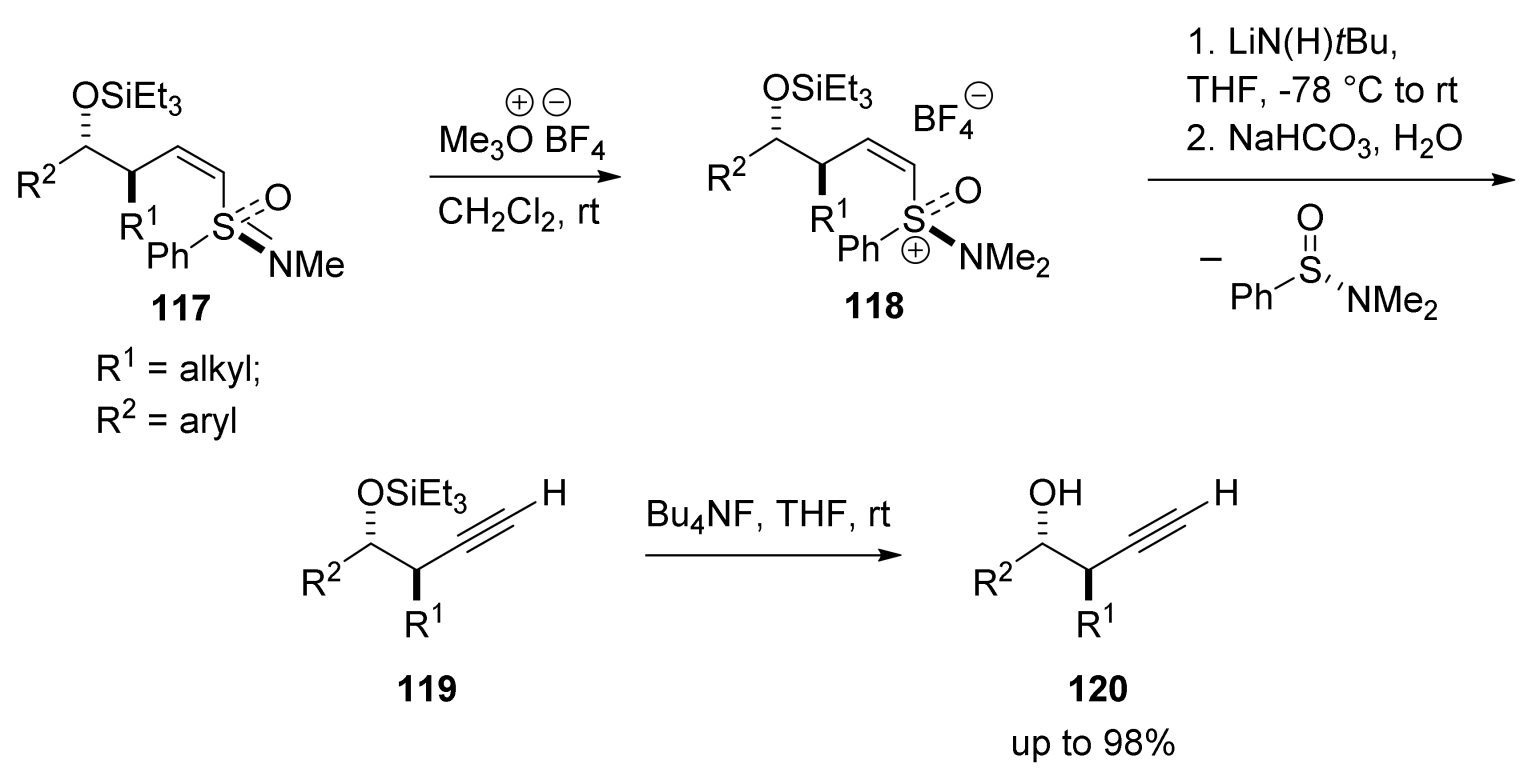
Synthesis of *anti*-homopropargylic alcohols.

Enantiomerically pure homopropargylic alcohols have frequently been used as precursors in natural product synthesis [56]. Gais and co-workers approached the asymmetric synthesis of *anti*-homopropargylic alcohols **120** through utilization of their sulfonimidoyl-substituted homoallylic alcohols **117** (Scheme 40).

The latter compounds were prepared through the addition of chiral sulfonimidoyl substituted bis(allyl)titanium complexes to unsaturated or highly branched aldehydes [57]. Methylation at the *N*-atom converted the sulfonimidoyl group on the alkenylsulfoximines **117** into a (dimethylamino)sulfoxonium group (in **118**), which would act as a better leaving group. The synthesis of the desired homopropargylic alcohols **120** was then completed by LiN(H)*t*Bu-mediated elimination of the alkenyl(dimethylamino)sulfoxonium salt **118**, followed by deprotection of the resulting silyl ether **119** [57].

**Scheme 41:**
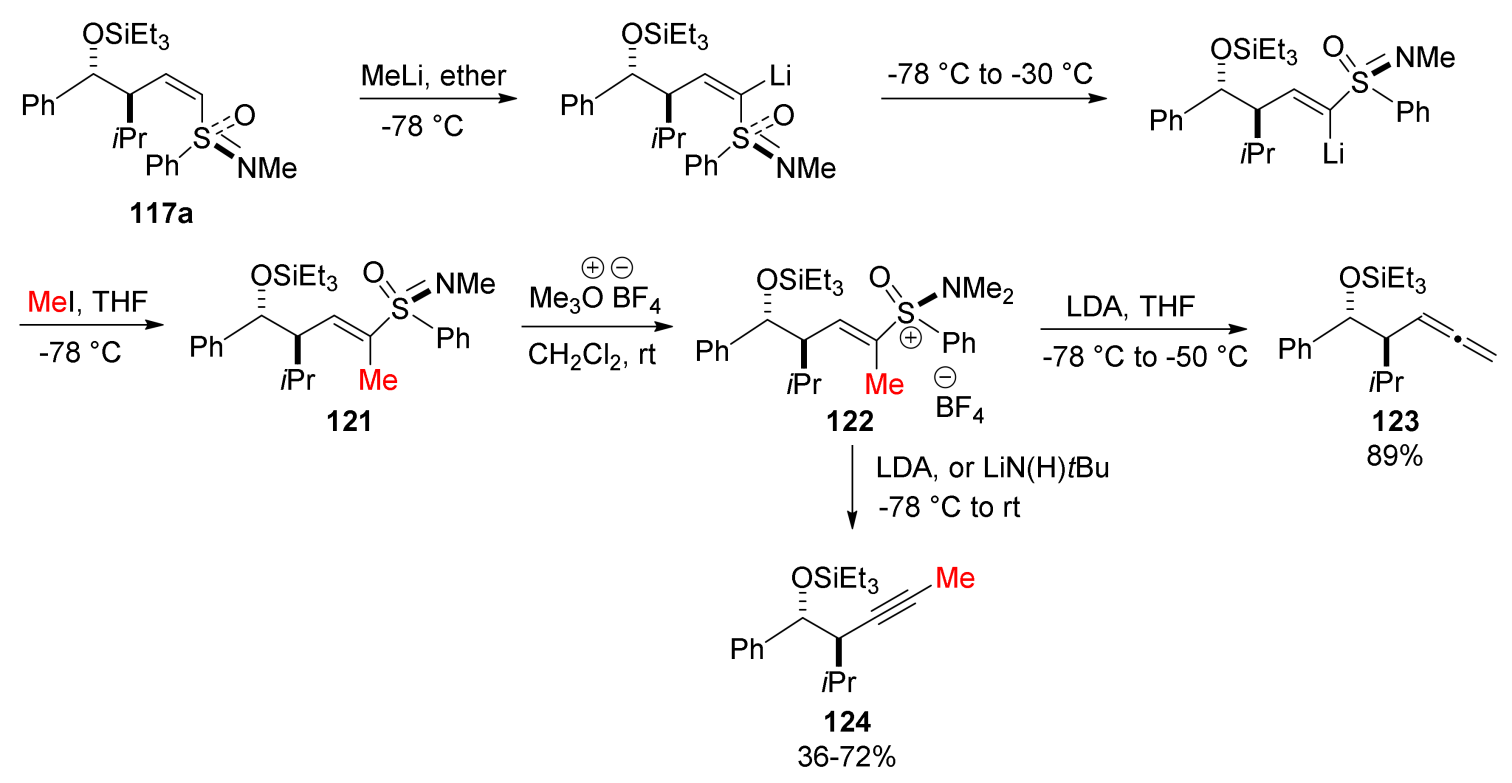
Synthesis of an internal homopropargylic alcohol and a homoallenylic alcohol derivative.

Facile lithiation of the alkenylsulfoximine **117a** at the *α*-position opened up access to nonterminal homopropargylic alcohols **124**. Introduction of a substituent to the *α*-position of **117a** afforded **121**. Elimination of the corresponding *α*-methylated alkenyl(dimethylamino)sulfoxonium salt **122** at different temperatures led to either enantiomerically pure *anti*-configured homoallenylic alcohol derivative **123** or nonterminal homopropargylic alcohol derivative **124**, both of which are synthetically useful motifs (Scheme 41) [57].

### 6.2. Asymmetric Synthesis of Unsaturated, Fused Bicyclic Proline Analogues 

**Scheme 42:**
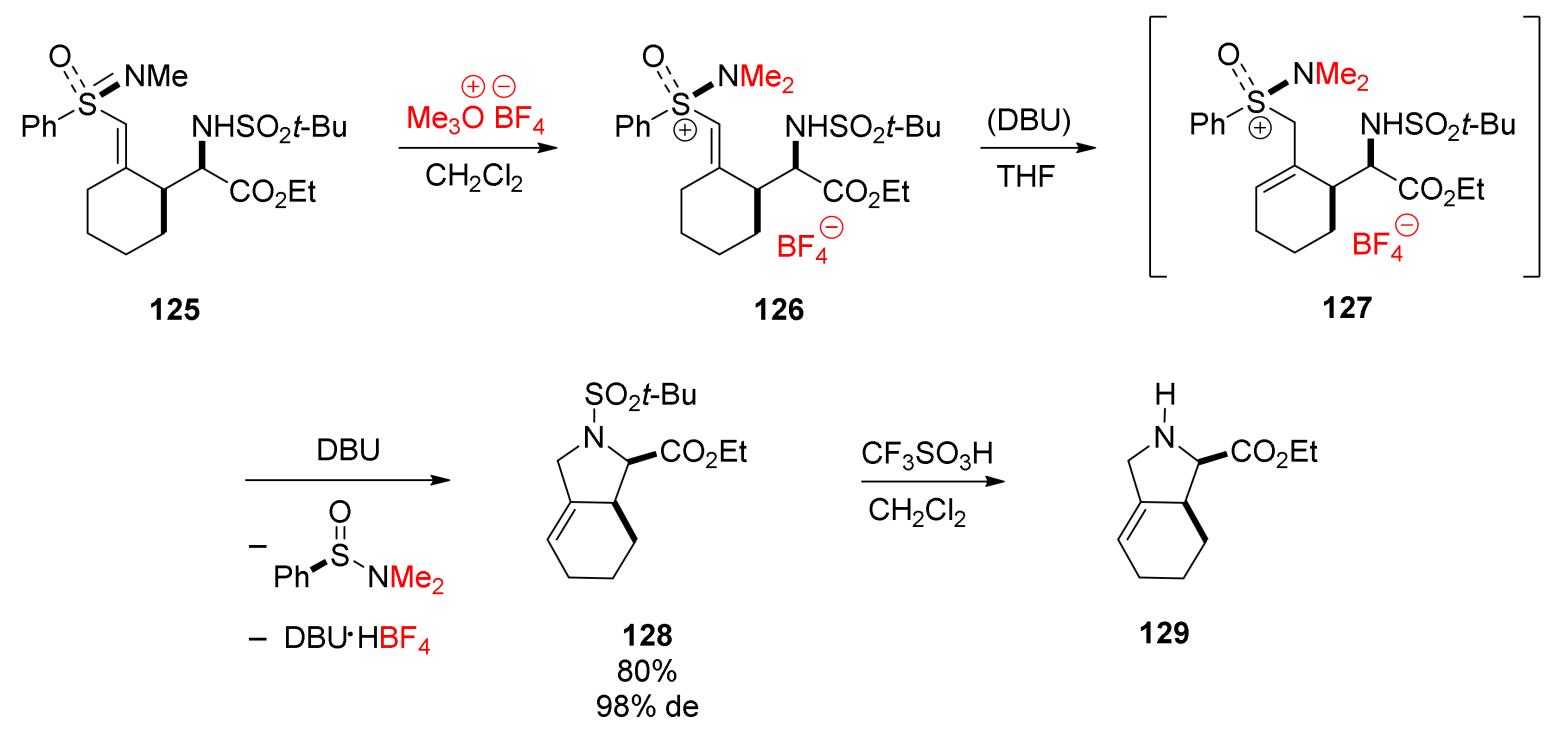
Synthesis of bicyclic proline analogues.

Gais et al. were also interested in the asymmetric synthesis of fused bicyclic proline analogues **129** due to their application as starting materials for the preparation of biologically active amino acids and peptidomimetics (Scheme 42) [22].

The required 4-amino 1-alkenyl sulfoximine **125** for methylation was prepared through alkylation of the enantiomerically pure cyclic bis(allylsulfoximine)titanium complex with *N*-*tert*-butylsulfonyl imino ethyl ester, proceeding with high regioselectivity and diastereoselectivity [22].

**Scheme 43:**
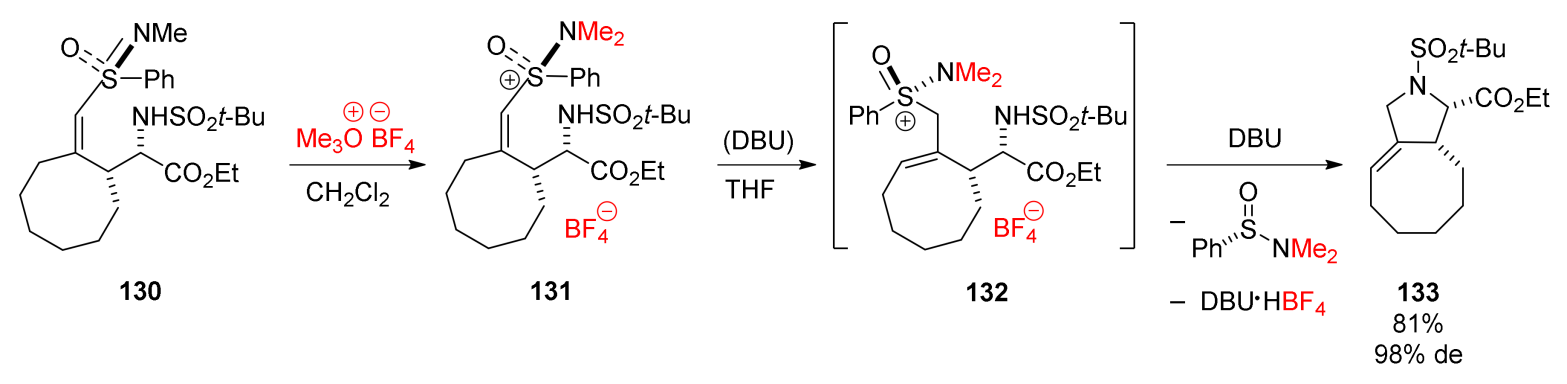
Synthesis of eight-membered bicyclic proline analogues.

Methylation of *N*-methyl sulfoximine **125** with Meerwein’s salt, to convert the sulfoximine into a good leaving group, provided the corresponding (dimethylamino)sulfoxonium salt **126** in excellent yield (≥95%). The novel migratory cyclization of the *δ*-amino alkenylsulfoxonium salt **126** was initiated by DBU. Isomerization of **126** to the corresponding allylic (dimethylamino)sulfoxonium salt **127**, followed by intramolecular substitution of the (dimethylamino)sulfoxonium group by the pendant sulfonamide group provided the pyrrolidine ring in **128**. Deprotection completed the synthesis of the enantiomerically and diastereomerically pure, unsaturated, fused bicyclic proline analogue **129**. The cyclization was not affected by the geometry of the alkenyl group in the vinylsulfoxonium salt **126**, and this method was also effective with an eight-membered carbocyclic ring **130** starting material (Scheme 43) [22].

### 6.3. Asymmetric Synthesis of 2,3-Dihydrofurans and Unsaturated Bicyclic Tetrahydrofurans.

**Scheme 44:**
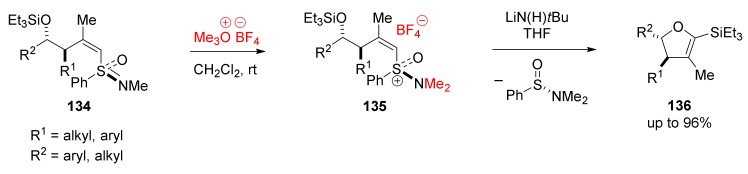
Asymmetric synthesis of monocyclic 2,3-dihydrofurans.

During their study of asymmetric synthesis of disubstituted homopropargylic alcohols **124**, Gais et al. observed that the *β*-Me-substituted alkenyl sulfoxonium salt **135** would give the 2,3-dihydrofuran as the product under the basic reaction conditions employed [57]. They pursued this line of enquiry and examined the reaction with different aryl and sterically bulky alkyl substituents at the 3-position [23]. Methylation of the acyclic *δ*-silyloxysubstituted alkenyl sulfoximines **134** and subsequent treatment with one equivalent of LiN(H)*t*-Bu smoothly gave the silyl-substituted monocyclic 2,3-dihydrofurans **136** in near quantitative yields (Scheme 44).

**Scheme 45:**
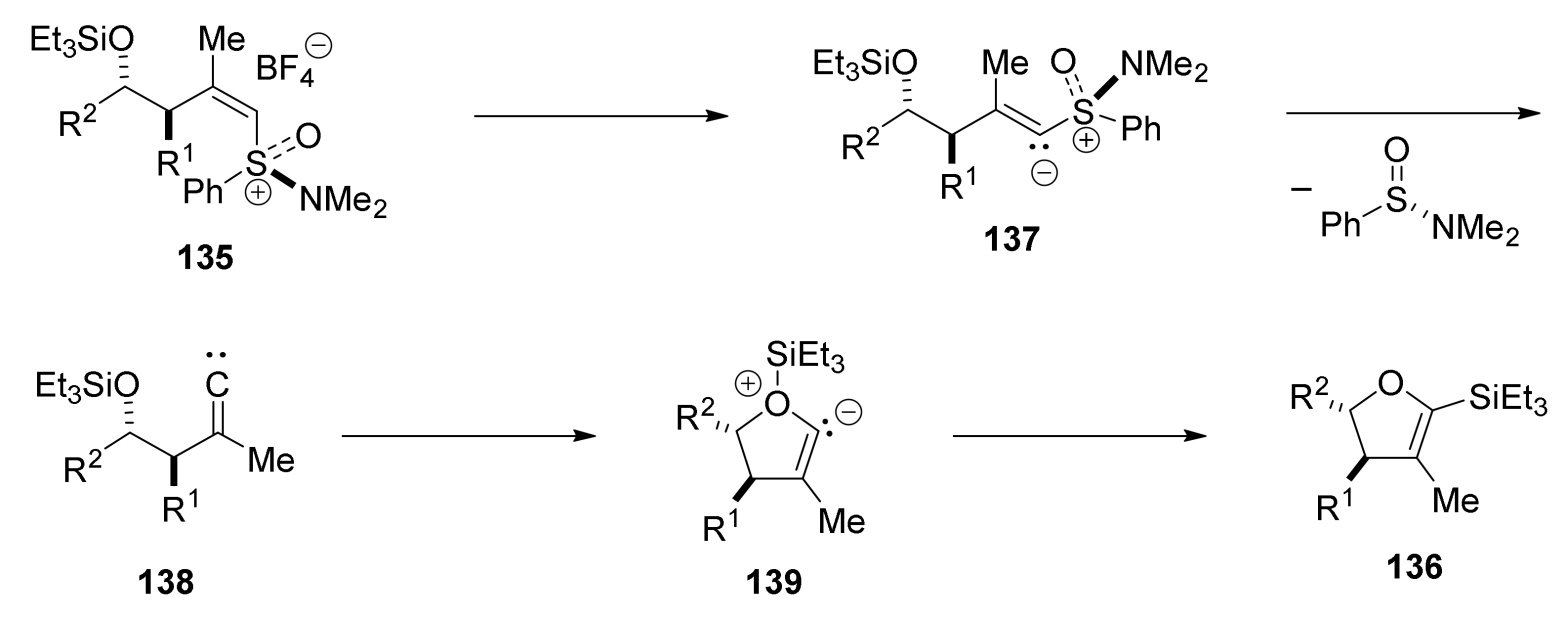
Proposed mechanism for the formation of 2,3-dihydrofurans.

Similarly, cyclic allylic sulfoximines were transformed to the corresponding enantiomerically pure bicyclic 2,3-dihydrofurans containing a six-, seven-, or eight-membered carbocyclic ring in high yields [23]. The authors proposed that the base effected *α*-elimination of the 1-alkenyl aminosulfoxonium salts **135** at higher temperatures, and that the resulting *β*-silyloxy alkylidene carbenes **138** underwent a 1,5-O,Si bond insertion followed by a 1,2-silyl migration to access the desired products **136** (Scheme 45) [23].

**Scheme 46:**
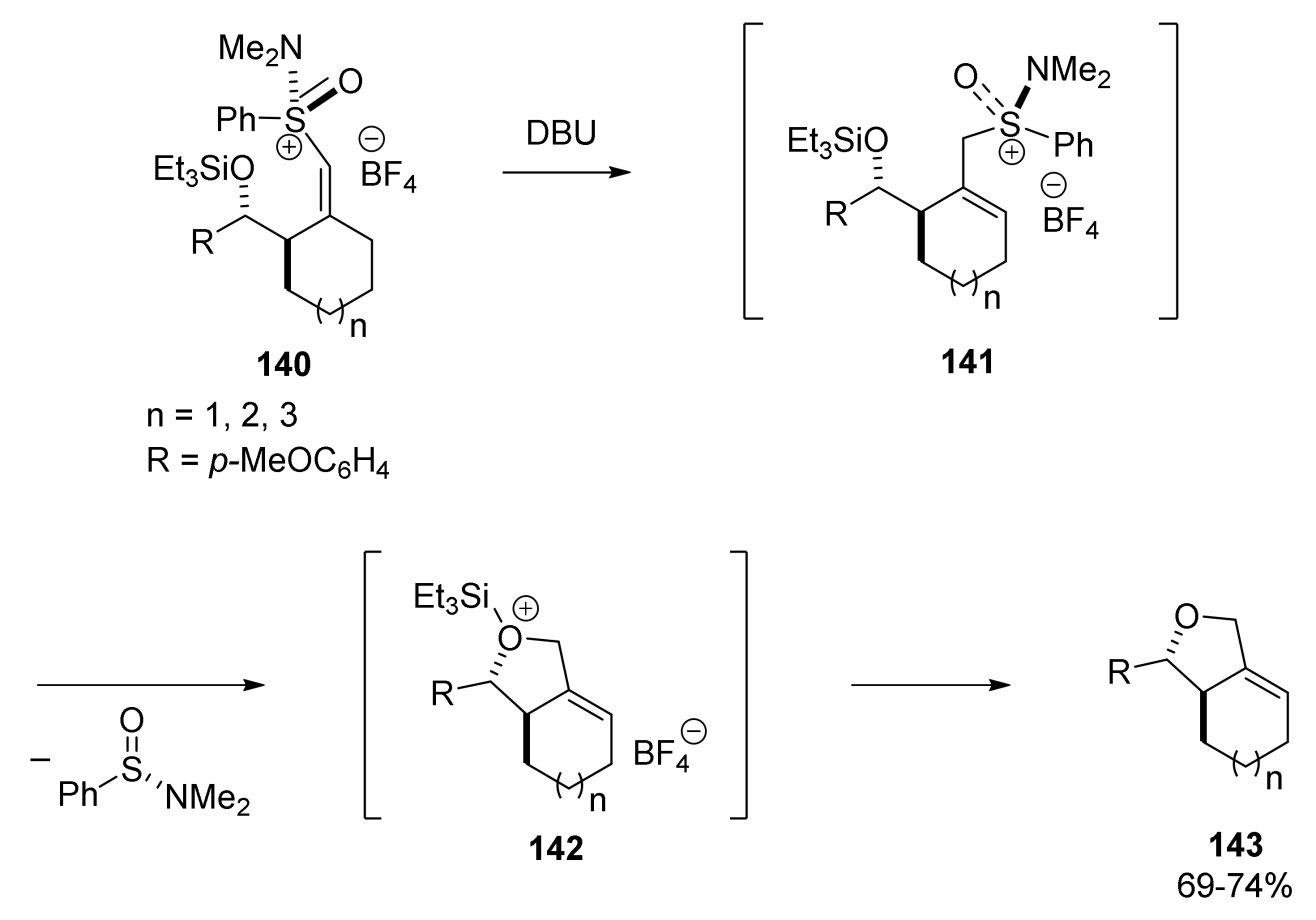
Asymmetric synthesis of unsaturated fused bicyclic tetrahydrofurans.

Inspired by their previous results whereby treatment of (sulfonylamino)alkyl-substituted 1-alkenyl aminosulfoxonium salts **126** with DBU furnished unsaturated fused bicyclic proline derivatives **129** via a novel migratory cyclization, Gais and his colleagues applied the same strategy to the asymmetric synthesis of unsaturated fused bicyclic tetrahydrofurans **143**. The latter compounds represent synthetically interesting skeletons found in many natural products [22,23]. Instead of an *α*-elimination, the weak base DBU facilitated the isomerization of the 1-alkenyl aminosulfoxonium salts **140** to the allylic aminosulfoxonium salts **141**. The allylic aminosulfoxonium salts **141** proceeded to undergo intramolecular substitution and desilylation to produce enantioenriched bicyclic tetrahydrofurans **143** of various ring sizes in good yields (Scheme 46) [23].

### 6.4. Asymmetric Synthesis of Unsaturated Prolines, β,γ-Dehydro Amino Acids, and Cyclopentanoid Keto Aminosulfoxonium Ylides

**Scheme 47:**
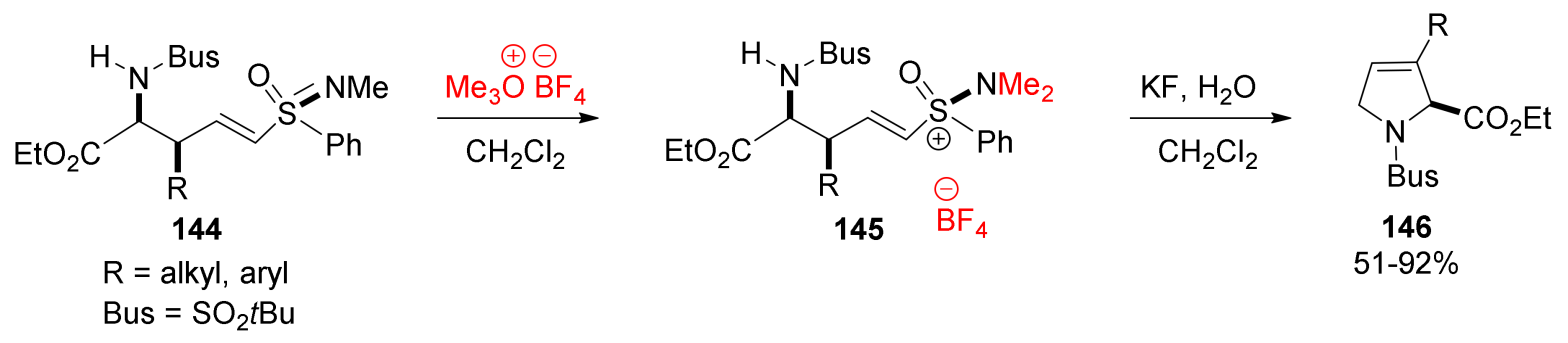
Asymmetric synthesis of 3,4-dehydro prolines.

Gais and co-workers extended their study on the asymmetric synthesis of proline derivatives **146** after they reported the formation of unsaturated bicyclic prolines **129** [22]. They applied a similar strategy but this time employing a F^−^ ion-mediated migratory cyclization rather than DBU, to deliver the desired monocyclic 3,4-unsaturated prolines **146** with ≥98% ee and in moderate to excellent yields (Scheme 47) [24]. The vinyl aminosulfoxonium salts **145** subjected to cyclization in the three-phase system were prepared by activation of the corresponding chiral vinylsulfoximines **144** through methylation with Meerwein’s salt. This procedure was successful with a sterically bulky substituent at the 3-position of the proline products **146 [24]**.

**Scheme 48:**
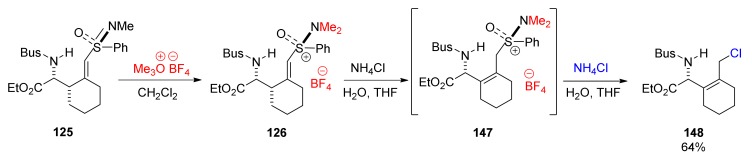
Asymmetric synthesis of cyclic *δ*-chloro-*β,γ*-dehydro amino acid.

As reported earlier, DBU-initiated migratory cyclization of the chiral vinyl aminosulfoxonium salts **126** would lead to bicyclic prolines **129** [22]. An accidental experiment involving treatment of the salts with NH_4_Cl, instead of DBU or KF in THF/water, resulted in an unexpected substitution product. The allyl chloride **148** was obtained in 64% yield (Scheme 48) [24]. Comparable yields of corresponding products were obtained in the case of treatment of acyclic vinyl aminosulfoxonium salts with NaCl or NH_4_Cl. The preference for migratory substitution over migratory cyclization of salts could be explained by the differences in nucleophilicity and basicity of the Cl^−^ and the F^−^ anions [24].

**Scheme 49:**
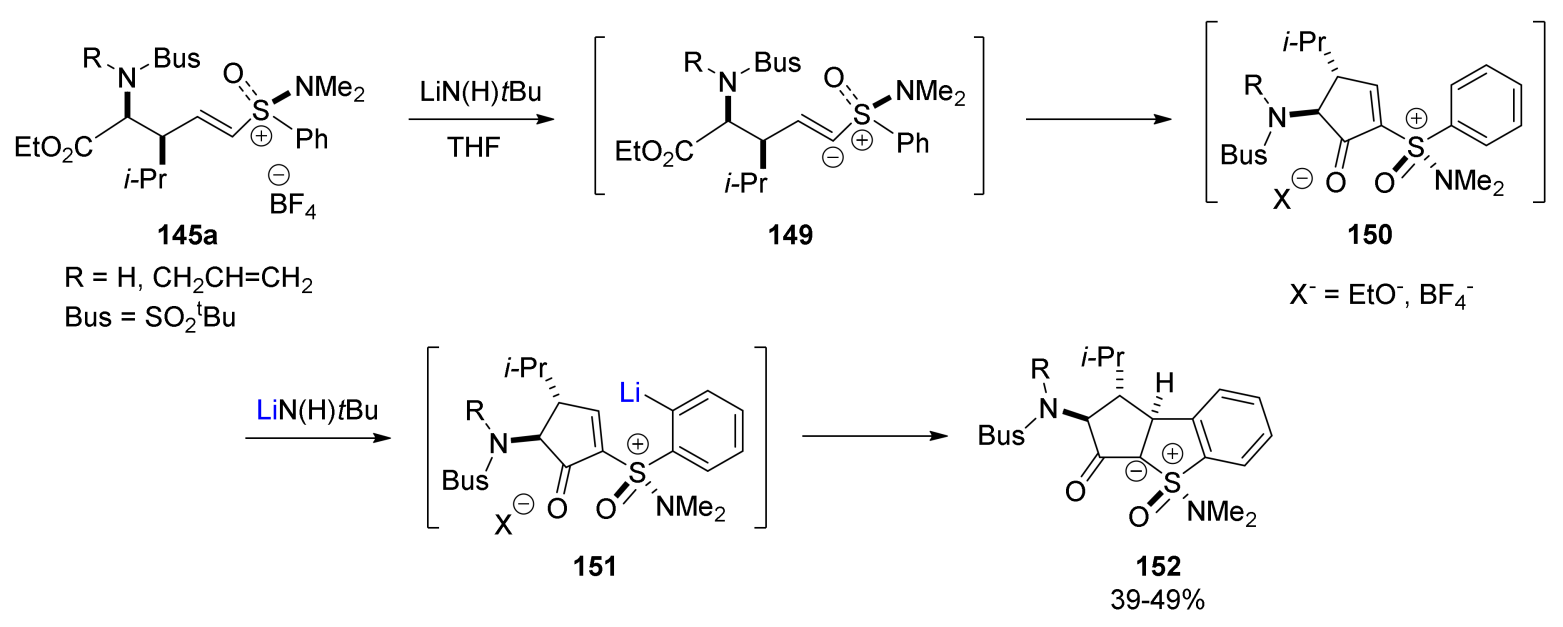
Synthesis of amino-substituted tricyclic ylides.

When the vinyl aminosulfoxonium salt **145a** was treated with strong base LiN(H)*t*-Bu in THF at low temperature, the tricyclic keto aminosulfoxonium ylide **152** was observed and isolated (Scheme 49) [24]. The vinyl aminosulfoxonium salt **145a** was most likely deprotonated by lithium amide to form the corresponding vinyl aminosulfoxonium ylide **149** initially. Subsequent cyclization of the ylide through the attack of the ylidic carbon atom on the ester group provided access to the cyclopentenone derivative **150**. *Ortho*-lithiation followed by a stereoselective intramolecular conjugate addition of the aryl group furnished novel tricyclic ylide **152 [24]**.

### 6.5. Diastereoselective Synthesis of γ-Lactones through Reaction of Enediolates with α,β-Unsaturated Sulfoxonium Salts

*γ*-Lactones are structural elements that are found in 10% of all natural products, including the paraconic acids, and an array of bicyclic and tricyclic ring systems (e.g., xanthatin) [58]. *γ*-Lactones possess interesting biological activity including strong antifungal, antibiotic, antitumor, antiviral, and *anti*-inflammatory activity, which suggests that they have potential as leads for pharmaceutical discovery [58,59,60,61]. Moreover, *γ*-lactones have been frequently used as synthetic intermediates for the assembly of complex molecules [62].

Kerrigan’s group investigated *γ*-lactone synthesis by studying the reaction of lithium enediolates **156** with *α*,*β*-unsaturated sulfoxonium salts **154** [63]. This approach complemented their previously reported one-pot methodology for the preparation of *γ*-lactones from sulfoxonium ylides and disubstituted ketenes, in that it mainly provided access to *γ*-lactones **155** bearing *α*- and *β*-tertiary stereogenic centers [63,64]. The sulfoxonium salt **154** was prepared by procedures previously described by Johnson and co-workers [19,20,21].

**Scheme 50:**
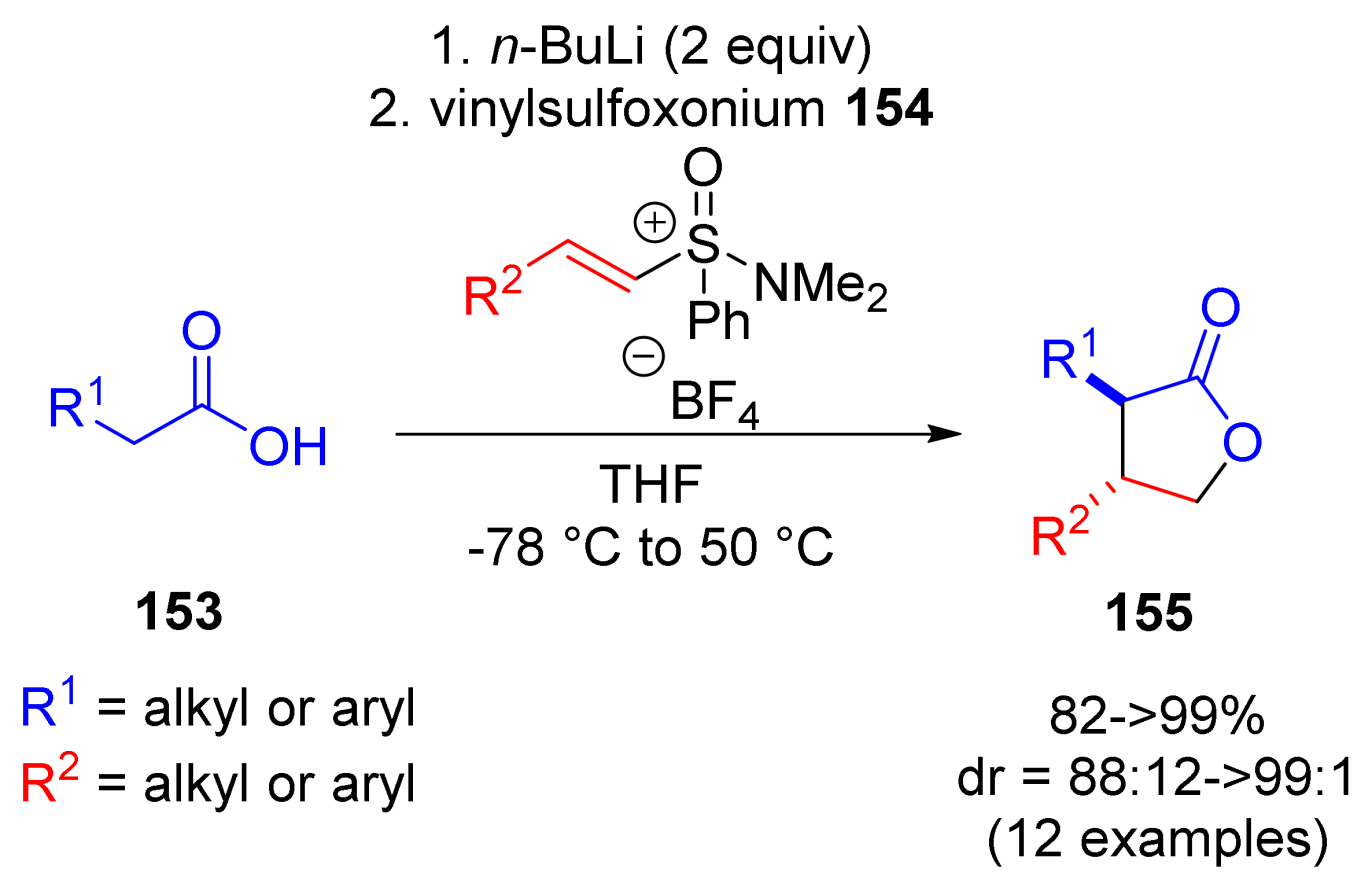
Synthesis of *γ*-lactones through reaction of enediolates with *α*,*β*-unsaturated sulfoxonium salts.

The reaction proved to be tolerant of a range of aryl substituents *α* to the carboxyl group of the carboxylic acid, including both electron-donating and electron-withdrawing substituents (Scheme 50). Impressively, even substitution at the 2-position on aryl substituents was tolerated without any significant effect on yield in reactions with styrenyl sulfoxonium salt **154a** (R^2^ = Ph). Good results were also achieved with the *i*-Pr-substituted vinyl sulfoxonium salt **154b** (R^2^ = *i*-Pr). Invariably, high diastereoselectivity (dr ≥ 88:12), favoring formation of the *trans*-diastereomer as the major isomer, was observed. Significantly, the reactions of lithium enediolates derived from *α*,*α*-disubstituted acetic acids were found to proceed smoothly to provide access to the desired *α*-quaternary center substituted *γ*-lactones, albeit with lower diastereoselectivity (dr 80:20) [63,64].

**Scheme 51:**
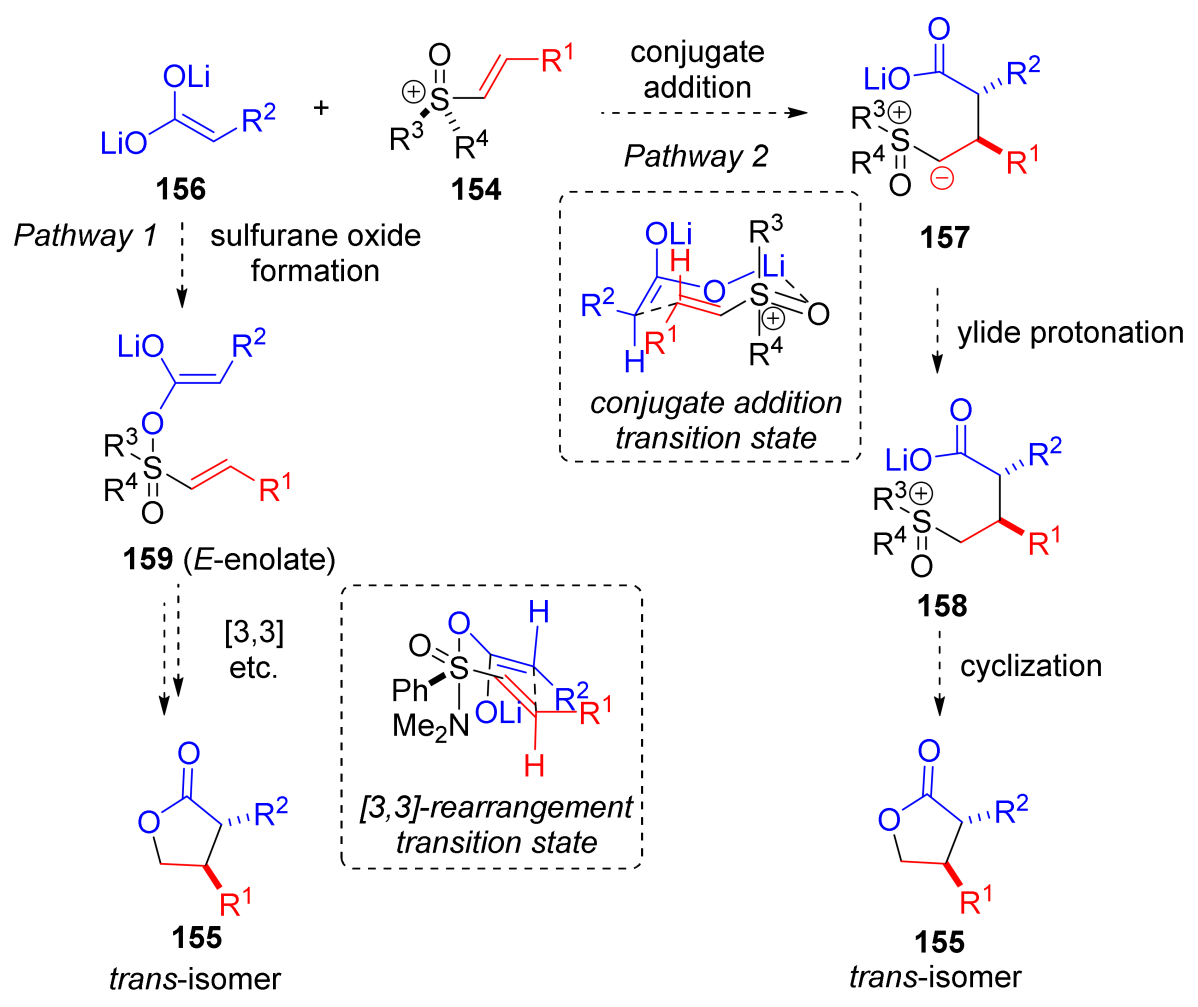
Possible reaction mechanisms for the formation of *γ*-lactone from vinylsulfoxonium salt.

Two possible mechanisms were proposed for the formation of *γ*-lactone **155**. In the first mechanism, Pathway 1 (Scheme 51), the lithium enediolate **156** adds to the sulfur of the vinylsulfoxonium salt **154** to give enolate intermediate **159** in stereoselective fashion as the *E*-isomer. Subsequent [3,3]-sigmatropic rearrangement, ylide protonation (by acid/lactone/intramolecular proton transfer), and 5-exo-tet cyclization would lead to the formation of lactone **155** as the *trans*-diastereomer [64,65]. This mechanism is consistent with the one that Kerrigan and co-workers have proposed for the formation of *γ*-lactones from sulfoxonium ylide, aldehydes, and ketenes in that both involve enolate **159** as a key intermediate [64,66]. In the second pathway, ylide intermediate **157** would be formed through conjugate addition of lithium enediolate **156** to the *β*-position of vinyl sulfoxonium **154** (Pathway 2, Scheme 51) [67]. Protonation of ylide **157** (by acid/lactone/intramolecular proton transfer) followed by cyclization would provide access to *γ*-lactone **155**. It was speculated that such a mechanism could account for the high *trans*-diastereoselectivity observed if a closed transition state (through Li chelation) was involved in the conjugate addition step (Scheme 51) [68,69]. Alternatively, the high *trans*-diastereoselectivity may be obtained through equilibration (reversible deprotonation-protonation) of the lactone product under the reaction conditions (presence of excess enediolate) to favor the more thermodynamically stable *trans*-isomer. Curiously, vinylsulfonium salts failed to undergo the desired cyclization reaction, while the reaction of vinylphosphonium salts stopped at an intermediate phosphonium ylide analogous to ylide **157** (Scheme 51).

### 6.6. Asymmetric Synthesis of γ-Lactones through Koga Amine-Controlled Addition of Enediolates to α,β-Unsaturated Sulfoxonium Salts

**Scheme 52:**
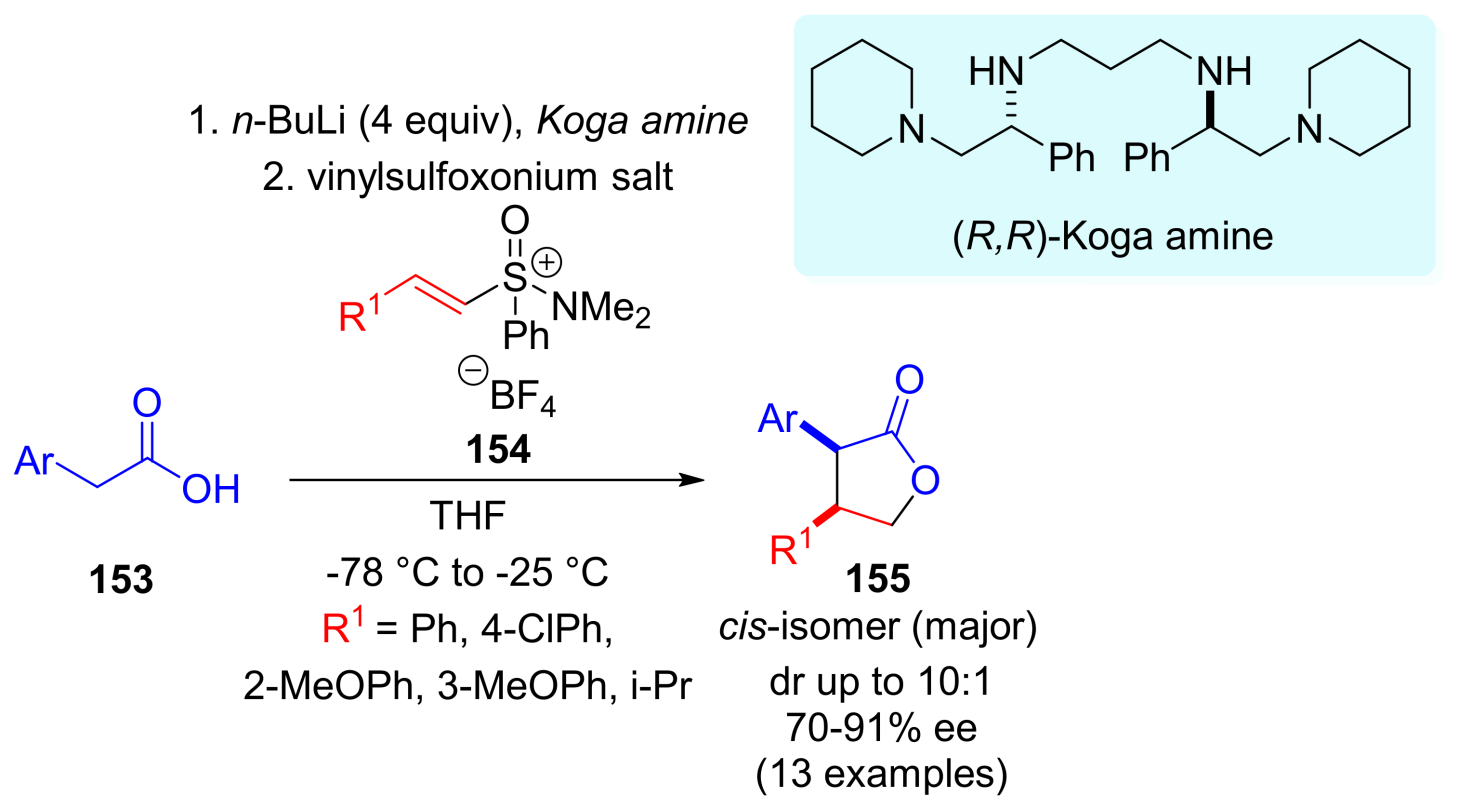
Asymmetric synthesis of *γ*-lactones through Koga amine-controlled reaction.

Subsequently, Kerrigan et al. were motivated to utilize chiral amine ligands to control the enantioselectivity of the reaction of lithium enediolates **156** with *α*,*β*-unsaturated sulfoxonium salts **154** [70]. *cis*-*γ*-Lactones **155** were obtained through a formal [3 + 2] cycloaddition process. The desired *γ*-lactones were formed in moderate to good yields (50–71%), with moderate to very good diastereoselectivity (dr up to 10:1), favoring the *cis*-isomer, and good to excellent enantioselectivity (Scheme 52). When enantioenriched aminosulfoxonium salts **154** were employed in combination with the appropriate enantiomer of Koga amine, a significant enhancement in asymmetric induction was observed. This increase in enantioselectivity was due to a match between the chirality of the sulfoxonium salt and that of the Koga amine [70,71].

**Scheme 53:**
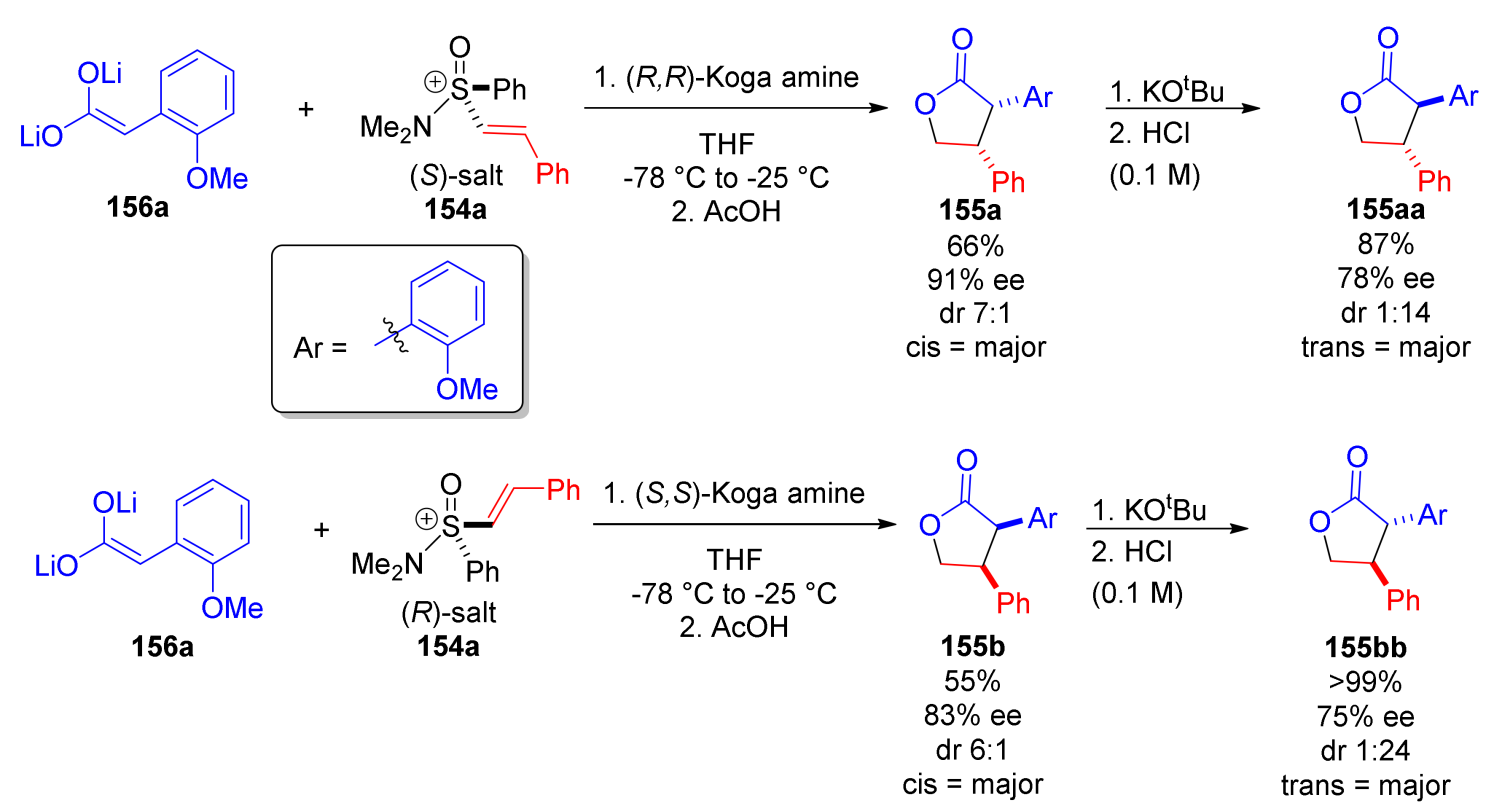
Asymmetric synthesis of all stereoisomers of **155a**.

The synthetic versatility of the technology was demonstrated by the simple conversion of enantioenriched **155a** and **155b** into the corresponding *trans*-isomers (**155aa** and **155bb**) through utilization of a base-mediated isomerization procedure (Scheme 53). As a result, all four stereoisomers of a given *γ*-lactone (**155a**) could be efficiently accessed with good to excellent diastereoselectivity (dr 6:1 to 24:1) and with good to excellent enantioselectivity (75−91% ee).

**Scheme 54:**
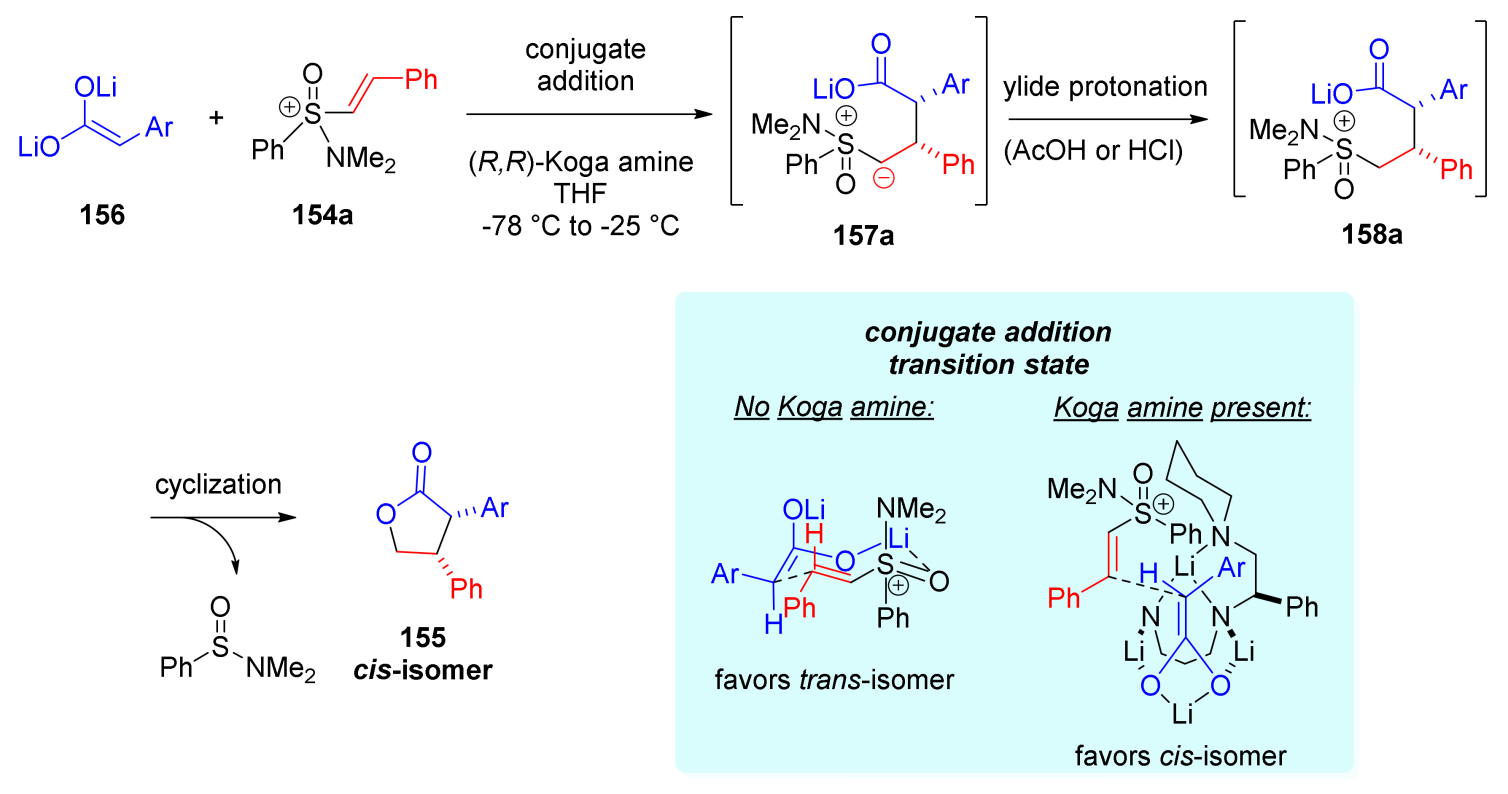
Proposed reaction mechanism.

The reaction most likely proceeds through a conjugate addition-ylide protonation–cyclization mechanism (Scheme 54). Ylide intermediate **157a** would be formed through conjugate addition of lithium enediolate **156** to the *β*-position of the vinyl sulfoxonium **154a** [67]. Subsequent protonation of ylide **157a** by added acetic acid would lead to the formation of intermediate **158a**, followed by cyclization, to provide *γ*-lactone **155**. The complete change in the sense of diastereoselectivity to favor the *cis*-isomer, in contrast to the stereochemical outcome of Kerrigan group’s earlier studies conducted in the absence of Koga amine, may be explained by the conjugate addition step proceeding through an open antiperiplanar transition state [63,68,69]. This differs from the previously proposed transition state for the reaction conducted in the absence of Koga amine where a closed transition state, involving lithium chelation of the sulfoxonium oxygen and enediolate, would lead to the *trans*-isomer dominating (Scheme 54) [63].

## 7. Conclusions

Vinylsulfonium and vinylsulfoxonium salts are classes of important, and valuable Michael acceptor reagents providing opportunities for the synthesis of an array of useful cyclic products. They are widely used in organic synthesis due to their unique capability. They generally play a double role in the reactions serving as an activating group, for nucleophilic addition *β* to the sulfur moiety, and then acting as a leaving group, enabling cyclization. These special salts possessing dual function are vital in enabling the streamlined synthesis of three to seven membered heterocycles, fused heterocycles, and other essential synthetic intermediates. Diastereoselective and enantioselective reactions of these salts have been recently developed through use of chiral reactant partners or chiral onium salts (substrate/chiral auxiliary control) or the use of chiral ligands (reagent control). These recent advances in asymmetric synthesis methodologies should lead to an increase in practical applications such as the synthesis of pharmaceutical, agrochemical, and biologically active compounds. While the groups of Aggarwal and others have exploited the chemistry of vinylsulfonium salts to a great extent in recent times, much work remains to be carried out in the exploration of the chemistry of the analogous vinylsulfoxonium salts. To enable such studies, improvements in methods for the synthesis of vinylsulfoxonium salts will first need to be achieved. In addition, the development of catalytic reactions involving vinylsulfonium and vinylsulfoxonium salts remains uncharted territory.
